# A Comprehensive Review on Upconversion Nanomaterials-Based Fluorescent Sensor for Environment, Biology, Food and Medicine Applications

**DOI:** 10.3390/bios12111036

**Published:** 2022-11-17

**Authors:** Wei Jiang, Jiaqi Yi, Xiaoshuang Li, Fei He, Na Niu, Ligang Chen

**Affiliations:** 1 College of Chemistry, Chemical Engineering and Resource Utilization, Key Laboratory of Forest Plant Ecology, Northeast Forestry University, 26 Hexing Road, Harbin 150040, China; 2 Key Laboratory of Superlight Materials and Surface Technology, Ministry of Education, College of Materials Science and Chemical Engineering, Harbin Engineering University, Harbin 150001, China

**Keywords:** upconversion nanoparticles, surface modification, environmental analysis, biological analysis, food and medicine analysis

## Abstract

Near-infrared-excited upconversion nanoparticles (UCNPs) have multicolor emissions, a low auto-fluorescence background, a high chemical stability, and a long fluorescence lifetime. The fluorescent probes based on UCNPs have achieved great success in the analysis of different samples. Here, we presented the research results of UCNPs probes utilized in analytical applications including environment, biology, food and medicine in the last five years; we also introduced the design and construction of upconversion optical sensing platforms. Future trends and challenges of the UCNPs used in the analytical field have also been discussed with particular emphasis.

## 1. Introduction

In 1959, Bloemberge et al. excited ZnS with 960 nm infrared and obtained its emission peak at 525 nm. Later, in 1961, Auzel observed two-photon excitation of fluorescence in Eu^3+^ doped crystals under high-power laser irradiation. Since then, the conjecture that energy may transfer between two rare-earth ions has been deeply rooted in the work of researchers. It was not until 1966, when phosphors doped with rare-earth ions were successfully synthesized, that the idea of upconversion luminescence was formally proposed. After more than 60 years of research. Lanthanide-doped upconversion nanoparticles (UCNPs) have shown high luminescence efficiency due to the long lifetime of the excited state, and they have been studied by researchers from all walks of life, gaining popularity in biomarkers, contrast agents, and other technologies. Upconversion luminescence, also known as anti-Stokes luminescence, is a particular luminescence phenomenon. The wavelength of the emitted light is less than that of the exciting light [1]. Specifically, the UCNPs luminescence mechanism is based on the following process: the luminescent center absorbs two or more low-energy photons, undergoes radiation-free decay to reach the excited state energy level, and thus returns to the ground state and releases a high-energy photon. In order to efficiently realize two-photon or multi-photon processes, the excited states of the luminescent centers need to have long energy level lifetimes. Moreover, the jumps between energy levels of rare-earth ions belong to forbidden f-f jumps with long energy level lifetimes. Therefore, the upconversion luminescence mechanism study also needs to focus on the energy level leap of rare-earth ions [2,3].

**Figure 1:**
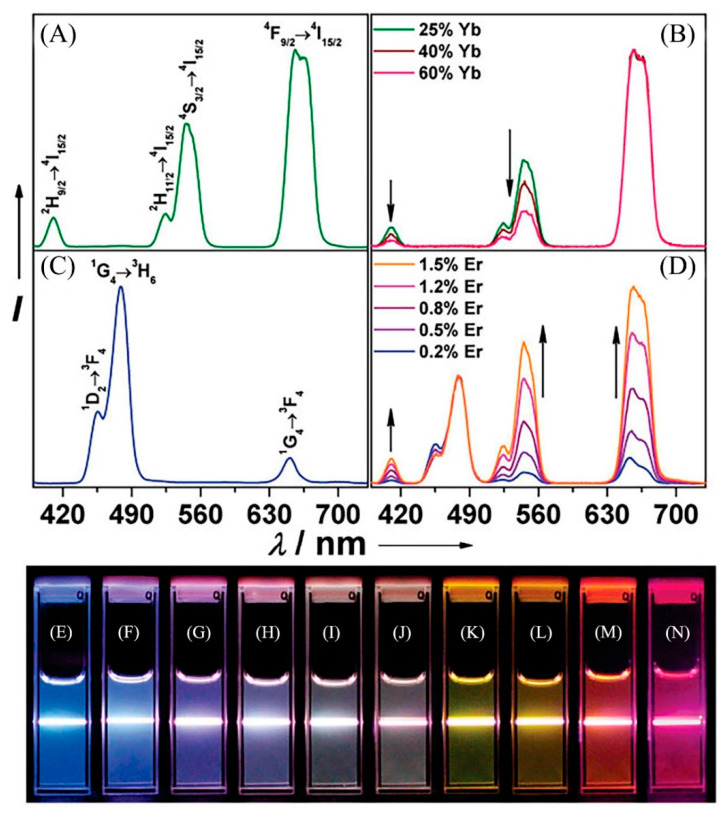
Room temperature upconversion emission spectra of (**A**) NaYF_4_: Yb, Er (18/2 mol%), (**B**) NaYF_4_: Yb, Tm (20/0.2 mol%), (**C**) NaYF_4_: Yb, Er (25–60/2 mol%), and (**D**) NaYF_4_: Yb, Tm, Er (20/0.2/0.2–1.5 mol%) particles in ethanol solutions (10 mM). The spectra in (**C**) and (**D**) were normalized to Er^3+^ 650 nm and Tm∙480 nm emissions, respectively. Compiled luminescent photos showing corresponding colloidal solutions of (**E**) NaYF_4_: Yb, Tm (20/0.2 mol%), (**F**–**J**) NaYF_4_: Yb, Tm, Er (20/0.2/0.2–1.5 mol%), and (**K**–**N**) NaYF_4_: Yb, Er (18–60/2 mol%). The samples were excited at 980 nm with a 600 mW diode laser. The photographs were taken with exposure times of 3.2 s for (**E**–**I**) and 10 s for (**M**,**N**). Reprinted with permission from [7], copyright 2008, American Chemical Society.

As we know, the UCNPs consist of a matrix, sensitizer, and activator. Some single-doped UCNPs do not contain the sensitizer. Among them, the matrix is the central part of the UCNPs. Many studies have proved that Y^3+^, La^3+^, and Lu^3+^ ions are more suitable for matrix materials. The ions that are incorporated into the matrix as luminescence centers are called activators. Rare-earth ions with abundant energy levels such as Pr^3+^, Nd^3+^, Sm^3+^, Tb^3+^, Ho^3+^, Er^3+^, Tm^3+^, etc., are commonly used as UCNPs activators. Moreover, increasing the doping concentration from the single doping activator UCNPs will cause a certain degree of luminescence of reduction [4,5,6]. In order to improve the luminescence efficiency, in the presence of the luminescence center, another rare-earth ion is doped with a high concentration, namely, the sensitizer. Unlike other trivalent rare-earth ions, Yb^3+^ has only one excited state, which does not reduce the luminescence properties of materials, due to concentration quenching, energy transfer, and other factors. Therefore, Yb^3+^ is the most commonly used sensitizer. In general, Yb^3+^-Er^3+^, Yb^3+^-Tm^3+^, Yb^3+^-Ho^3+^ ion pairs double-doped UCNPs have high luminescence efficiency, and the doping of different proportions of Yb ions affects the luminescence intensity and emission peak position [7] (Figure 1), becoming the focus of research in recent years.

**Figure 2:**
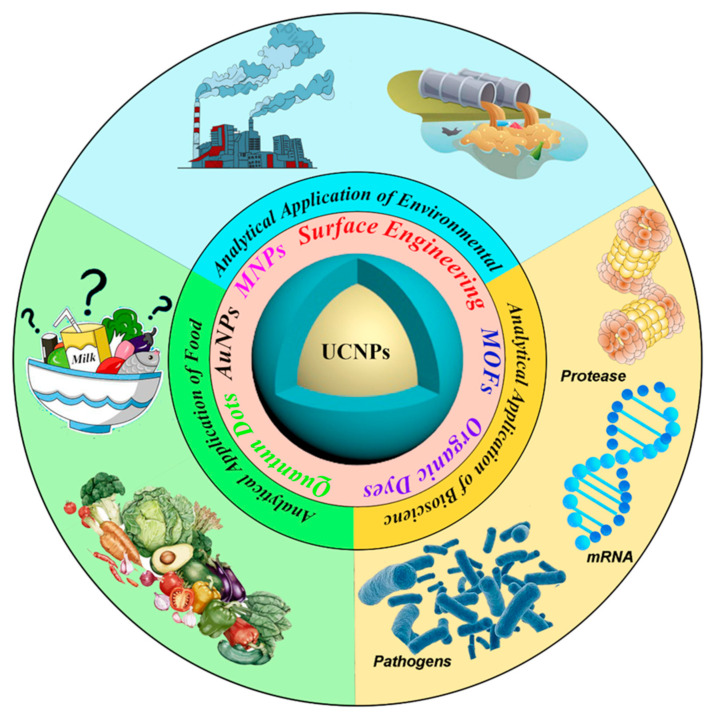
Schematic illustration of the fluorescent characteristics and sensing application of the UCNPs.

Although Atomic Absorption Spectrometry (AAS) [8,9], High-Performance Liquid Chromatography (HPLC) [10,11] or Mass Spectrum [12] and other instrumental analytical methods have been widely used in the analysis of metal ion, liquid organic pollutant or other substances, their expensive analytical costs and limitations in real-time detection are determined by the environment in which they are used. In contrast, fluorescence methods such as organic dyes [13], quantum dots (QDs) [14], metal complexes [15] offer the advantages of simplicity of operation, high stability, rapidity and accuracy, and the possibility of real-time detection. Among them are UCNPs, with their specific light-emitting pattern forming their unique characteristics, such as narrow emission bandwidths, large anti-Stokes shift, multicolor emission, low auto-fluorescence background, long lifetime, and high penetration stability [16]. A large number of researchers have found that these properties of the UCNPs can be put to excellent use in analytical application studies. Moreover, UCNPs have low toxicity and good chemical stability, which can make up for the shortcomings of the above-mentioned fluorescent materials in analytical applications. More importantly, the UCNPs are easily functionalized and can be applied in various fields. In this paper, we will detail the contribution of UCNPs applied to chemical analysis. Furthermore, we attempt to summarize the fluorescent characteristics and sensing applications of UCNPs in systematic mean (Figure 2).

This review provides an overview of recent advances in the design and application of UCNPs, focusing on four distinct areas including environmental analysis, biological analysis, food analysis, and medicine analysis. It covers sensors and assays mostly based on the UCNPs with a small size. We first introduce the synthesis of rare-earth-doped UCNPs and the surface modification processes, then we review sensing processes mechanisms and finally focus on introducing general sensing schemes for UCNPs-based chemo-sensors and biosensors.

## 2. Design of the UCNPs Analysis Nanoplatform

The design of the UCNPs nanoplatforms is targeted at sensing applications. So, the synthesis of the UCNPs and the construction and working mechanism of the UCNPs nanoplatforms are a prerequisite for the development of sensing schemes.

### 2.1. Synthesis of the UCNPs

The different composition, crystalline shape, shape, and size of nanoparticles can affect their optical properties and potential utilization in applications in fluorescence analysis or other research fields. Therefore, its synthesis methods have received much attention. In this paper, we will introduce several commonly used synthesis methods such as high-temperature pyrolysis, hydrothermal synthesis, and solvothermal methods, etc. Here, we focus more on applying the UCNPs in the analysis field, so in this chapter, we describe more synthetic methods used in recent years of applications. Researchers can choose different synthesis methods for the preparation of the UCNPs according to the diverse needs of the application (Table 1).

#### 2.1.1. High-Temperature Decomposition Method

High-temperature pyrolysis is one of the most common methods to synthesize rare-earth UCNPs. The process involves the rare-earth precursors being dissolved in a high boiling point organic solvent and then treating them at high temperature in the presence of a surfactant for a certain period to obtain rare-earth nanoparticles with uniform morphology and controlled size. The rare-earth trifluoroacetic acid or rare-earth chloride are used as precursors, and oleic acid (OA), oleylamines, or octadecenoic with long chains and polar ends are commonly used as surfactants [17,18,19]. Using trifluoroacetate as the precursor, Chow et al. [20] used oleylamine solvent to prepare the 10 nm NaYF_4_: Yb, Er. Furthermore, different reaction times, reaction reagent volume, and different reaction temperatures will affect the size and morphology of the UCNPs. While the incorporation of oily surfactants in the synthesis process poses difficulties for the application of UCNPs in analytical or biological fields, research on surface modification or functionalized UCNPs has become popular and important.

#### 2.1.2. Hydrothermal Method

In contrast, the synthesis conditions of the hydrothermal method are more uncomplicated. The solubility and reaction rate of the inorganic precursors are increased in a solvent system by controlling a suitable temperature and a vapor pressure above a solvent system’s critical value. Then, the method of preparing UCNPs by subsequent crystallization of the dissolved precursor ions from the solvent, under high temperature and pressure conditions, is conducted. This method uses a low-cost environmental solvent, and the reaction conditions are easy to control. In the process of hydrothermal synthesis, choosing the appropriate additive with specific functional groups of nanometer particle morphology and size of the control counts a great deal. Li et al. [21] proposed a liquid–solid liquid-phase method to synthesize high-quality rare-earth fluoride nanoparticles. Many kinds of nano-micron particles with different morphology and structure have been prepared by the hydrothermal method. The additives can be used as complexing agents and structure guiding agents in the process of the formation of nano-particles. Usually, the organic additives used in the synthesis of rare-earth nanoparticles include (OA) [22,23,24], ethylenediaminetetraacetic acid (EDTA) [25,26,27], cetyltrimethylammonuim bromide (CTAB) [28,29], trisodium citrate [30,31] and so on.

#### 2.1.3. Solvothermal Method

In solvothermal methods, simple hydrothermal methods show great potential for preparing one-dimensional rare-earth ion–doped nanowires, nanorods, or nanotubes, especially for preparing rare-earth hydroxides, when no organic additives or templates are used. For example, hydroxide nanowires, nanotubes, and nanorods can be selectively synthesized in a water system of rare-earth ions and NaOH (or KOH, NH_3_·H_2_O) under specified temperature and pH conditions [32,33]. In order to fine-tune the morphology and size of the target product, the most effective and direct strategy is to add appropriate organic active agents. On the one hand, free ion concentration affects monomer concentration and growth kinetics, while the actual free ion concentration depends on the coordination between metal ions and hydrophilic ligands. On the other hand, the selective adsorption of organic ligands on the crystal surface is beneficial to obtain controllable crystal morphology. Organic active agents include hydrophilicity and hydrophobicity. Commonly used hydrophilic surfactants include citric acid (Cit^3+^), EDTA, polyvinylpyrrolidone (PVP) [34], CTAB, polyvinylidene (PEI) [35], polyacrylic acid (PAA) [36,37], polyphosphoric acrylate (P-PAA) and 2-aminoethyl dihydro phosphates (APE). Zhang et al. [38] used OA as a surfactant to produce homogeneous NaYF_4_ nanorods, nanorods, and flower-shaped nanorods under different reaction conditions. In addition, OA as a surfactant can also be used to prepare rare-earth-doped nanoparticles with controllable morphology and size [39].

**Table 1:** Design of the UCNPs analysis nanoplatform.

Synthetic Methods	UCNPs	Precursors	Surfactant	Morphology	Size	Ref.
High-Temperature Decomposition Method	NaYF_4_: Yb/Er	RE(CF_3_COO)_3_	OA, ODE	Spherical	10 nm–30 nm	[40]
	NaLuF_4_: Yb, Er	RE(CF_3_COO)_3_		Ship-in-a-bottle	80 nm–240 nm	[41]
	NaGdF_4_: Yc, Er					
	LiYF_4_: Yd, Er					
	NaYF_4_: Yb, Er	RE(CF_3_COO)_3_	Oleylamine	Hexagonal	10.5 nm	[20]
	NaYF_4_: Yb, Tm					
	NaYF_4_: Yb, Er/Tm		OA, ODE	Large spheres	37.9 nm	[42]
				Small spheres	14.0 nm	
				Rods	Length = 60.1 nm, Width = 21.5 nm	
	β-NaYF_4_: Yb, Er/Tm	NH_4_F	OA, ODE	Nanoplates and	30 nm × 30 nm × 45 nm	[43]
				Nanospheres	30 nm	
				Nanoellipses	17 nm–22 nm	
Hydrothermal Method	NaYF_4_: Yb^3+^, Er^3+^	NaBF_4_	OA	pH = 7 Microtubes	14.46 μm	[44]
				pH = 9, Microrods and nanorods	10.65/0.87 μm	
				pH = 11, Microtubes	7.90 μm	
		NH_4_F		pH = 7, Submicrorods	5.53 μm	
				pH = 9, Submicrorods	4.07 μm	
				pH = 11, Submicrorods	0.48 μm	
		NaF		pH = 7, Microtubes	5.76 μm	
				pH = 9, Submicrorods	0.86 μm	
				pH = 11, Nanorods	0.65 μm	
	NaYF_4_: Yb^3+^, Er^3+^		OA, OH^−^	Nanobranches	1.5 μm	[45]
	β-NaLuF_4_	NaF	Aitric acid (AC)	AC = 2 mmol, regular hexagonal phase microdisks	Height: 0.79 μm Diameter: 7.58 μm	[46]
				AC = 3 mmol, short hexagonal phase microprisms	Height: 2.12 μm Diameter: 8.51 μm	
				AC = 8 mmol, hexagonal phase microtubes with hollow	Height: 9.47 μm Diameter: 1.88 μm	
	NaGdF_4_: Yb^3+^, Er^3+^	NaF	CTAB	Flower-like assemblies	200 nm–250 nm	[47]
Solvothermal Method	NaYF_4_: Yb, Er@NaYF_4_	NH_4_F	OA, ODE	Hexagonal nanoparticles	9 nm	[32]
	NaMF_4_	NaF, M(NO_3_)_3_	OA, OH^−^	Uniformly hexagonal nanotubes	Length: 500 nm Diameter: 250 nm	[38]

### 2.2. Surface Modification Strategies for the UCNPs

As noted above, our prepared UCNPs are dispersed in organic solvents such as cyclohexane and toluene due to the surface coating by OA or oleylamine, which can cause difficulties in subsequent studies. For example, as anti-Stokes fluorescent materials with near-infrared excitation and visible light emission, the UCNPs hydrophobic surfaces need to be converted to hydrophilic surfaces for analytical assays to be performed, then to graft-specific responsive, functional groups on the hydrophilic surface for the needs of the analyte. The hydrophilic process of the UCNPs was divided into ligand-free conversion and ligand modification. In ligand-free modifications, a good example is the acid treatment method used by Nicoleta Bogdan et al. [48], whereby Oleate-capped Ln-UCNPs were dispersed in an aqueous solution, and the pH was adjusted to 4 with HCL (0.1 mol L^−1^) to protonate the carboxylic acid group of the OA ligand (to produce OA). The UCNPs were obtained by extraction of the dispersed in water, while according to the study, the integrated UCNPs red-green emission ratio of Ln-UCNPs dispersed in toluene differs somewhat from that of Ln-UCNPs spread in water, which can be adjusted depending on the needs by adjusting the pH or by taking isotopic electronic isotopic [49]. The proposed scheme for the acid–base reaction occurring at the surface of the Ln-UCNP is as follows [48]:

(1) $$\mathrm{LnOA}\frac{\mathrm{HCl}}{\mathrm{pH}4}\to \mathrm{LnOH}+\mathrm{OAH}\to \;\left[{\mathrm{LnOH}}_{2}{}^{+}\right]\cdots {\mathrm{Cl}}^{-}$$

(2) $$\mathrm{LnOA}\frac{\mathrm{HCl}}{\mathrm{pH}4}\to \mathrm{LnOH}+\mathrm{OAH}\vec{\mathrm{pH}7.4}\left[{\mathrm{LnO}}^{-}\right]\cdots H_{3}O^{+}$$

In addition to the “ligand-free hydrophilic modification” process described above, ligand modification can be divided into organic ligands (-NH_2_, -COOH) and inorganic ligands (UCNPs@SiO_2_). The typical method for conversion of compounding of UCNPs@SiO_2_ is Stober’s reverse microemulsion method [50]. The approach is to form a water-in-oil microemulsion by adding the surfactant Igepal CO-520. After stirring, Igepal CO-520 and ammonium hydroxide were added again. Ultrasonication was performed to distribute the nanocrystals within the micelles uniformly. After adding TEOS and stirring for two days, organic solvents such as acetone were added to precipitate and separate the nanoparticles. Another surface silanization method is by adding ethyl orthosilicate dropwise (TEOS), at 70 °C, under alkaline conditions after 12 h of emulsification by adding the surfactant CTAB [51]. Different thickness of the silica shell (3–5 nm) affects the luminescence of the UCNPs to some extent, and the thickness of the shell layer can be controlled according to the application’s requirements. Although the coated silica can make the UCNPs surface hydrophilic, it does not provide the functional groups which the analyzed application process is coupled. Functionalization of the UCNPs organic ligands based on encapsulated silica is an essential part of the design of the UCNPs analysis platforms.

Table 2 illustrates some of the commonly used amination, carboxylation, and other surface modification UCNPs studies, usually the synthesis of amination UCNPs based on silylation, which is commonly performed by UCNPs of APTES or PEI grafting. In contrast, carboxylation sometimes does not require the process of silylation. However, some experiments will also be activated after the amination UCNPs and then connected to the carboxyl group. The table mentions some bioimaging or drug delivery (therapeutic) processes on the functionalization of UCNPs is hoped to bring analytical work. The functionalization of UCNPs for some bioimaging or drug-loaded (therapeutic) processes mentioned in the table is intended to inspire targeted functionalized UCNPs in some specific environments, such as He et al. [52] combined two representative biocompatible PCL and hydrophilic HPG on the UCNPs surface. Similarly, to improve the aggregation, precipitation, or degradation of the UCNPs in acidic, alkaline, or high ionic strength media, Markl et al. [32] designed self-assembling phospholipid bilayers (PLMs) functionalization NaYF_4_: Yb, Er, which can prevent instability in phosphate-buffered solution effectively, and it is a better application for in vivo analysis of organisms.

### 2.3. Construction of the UCNPs Nano-Platforms

**Figure 3:**
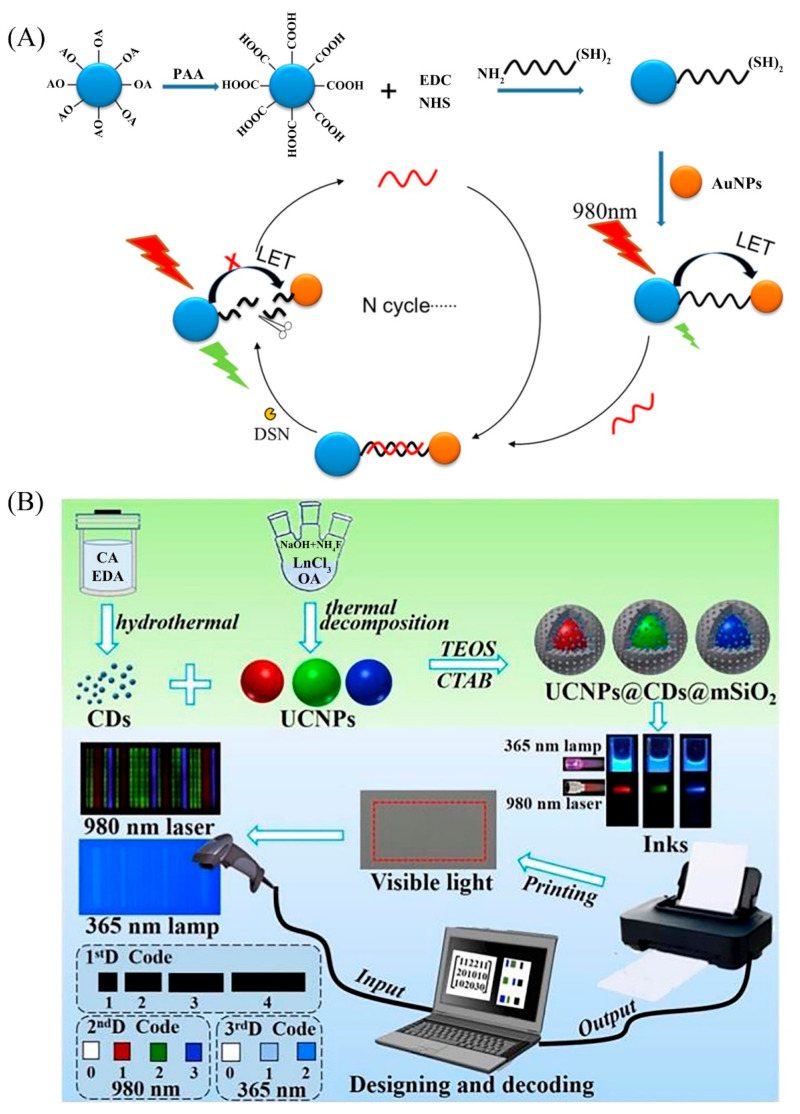
Schematic illustration based on the UCNPs analysis platform construction. (**A**) Schematic representation of UCNPs-AuNPs detection of miRNA155. (**B**) Schematic of both synthesis details and dual-mode fluorescence/color properties of sandwiched UCNPs@CDs@mSiO_2_ Core-Shell nanohybrids for anti-counterfeiting barcodes. Reprinted with permission from [33,56]. Copyright 2019, Elsevier and 2019, American Chemical Society.

It is well known that the UCNPs have a significant anti-Stokes shift and brilliant biocompatibility. While depending on the choice of the analyte, sometimes NaLnF_4_: Yb, Er (Tm) or another alone cannot achieve the purpose. However, the addition of two or more nanomaterials or biomaterials can help us achieve the purpose of analysis. For example, the absorption wave of gold nanoparticles overlaps with the emission spectrum of the UCNPs, which will affect the luminescence of part of the UCNPs [33]. Furthermore, gold nanoparticles are easy to modify the surface functional groups, so when analyzing different targets, fluorescence analysis can be carried out according to the different effects of the functional groups without affecting the fluorescence of the UCNPs. At the same time, in order to circumvent the problems of low-fluorescence resonance energy transfer (FRET) luminescence efficiency and low sensor sensitivity, targeted modification of the UCNPs has been an essential part of designing sensing platforms and is widely used in the analysis of metal ions and organic reagents as well [53,54]. In addition, also used is the coupling of biological elements such as antibodies, aptamers, and cDNAs using UCNPs and synergistic effects with carbon dots and graphene oxide quantum dots to analyze and detect microorganisms or small molecules such as fungal toxins [55,56,57] (Figure 3).

With the continuous optimization of magnetic separation technology, when the UCNPs are applied to the analysis of a specific environment, magnetic nanoparticles such as Fe_3_O_4_ synergize with them at the end of the analysis to facilitate the separation of the probe and the substance to be measured and facilitate the integration of enrichment and detection. Furthermore, the synergistic effect of some particles is to enhance the upconversion luminescence intensity and thus improve the detection efficiency, such as doping core–shell NaGdF_4_: Yb, Er@NaYF_4_: Yb^3+^, fluorescence enhancement more than NaLnF_4_: Yb, Er 1.6 times [58]. In addition, molecularly imprinted polymers (MIPs) molecular imprinting techniques have been combined with UCNPs to improve selectivity by exploiting the specific adsorption sites of molecularly imprinted polymers, thereby enhancing detection efficiency [59,60,61]. Apart from these, organic dyes [62] and fluorescein [63] are also used to form nano-fluorescent probes in synergy with UCNPs, and the applicable fluorescein is selected according to some unique properties of different substances to be measured. For example, in order to design a fluorescent probe for the detection of cysteine, Guan’s group [64] used probe 5 (6)-carboxy fluorescein-O, O′-diacrylate, which has a sensitive response to cysteine (Cys), combined with the effect on the position of the upconverted emission peak to achieve the purpose of analysis.

The design of the UCNPs nano-fluorescent probes for analytical assays has become a topical issue today. The selection of suitable structures of synergistic substances according to the requirements is still a challenging task that requires a lot of exploratory work researchers to subsequently develop innovative UCNPs fluorescent probes.

**Table 2:** Upconversion modifications and surface functionalization.

The UCNPs	Organic Ligands	Modified Material	Modified Purpose	Fluorescence Sensing Platform	Applications	Ref.
NaYF_4_: Yb, Tm	NH_2_-	3-aminopropyltriethoxysilane (APTES)	Coupling with aptamer	NaYF_4_: Yb, Tm-NH_2_/aptamer and SYBR Green-I	Oxytetracycline detecting	[65]
NaYF_4_: Yb, Tm		APTES	Coupling with aptamer	NaYF_4_: Yb, Tm-NH_2_@ Molecularly Imprinted Polymer-aptamer	Enrofloxacin detecting	[66]
NaYF_4_: Yb, Er@NaGdF_4_		APTES	Activate drug delivery	NaYF_4_: Yb, Er@NaGdF_4_	Intracellular imaging	[67]
NaYF_4_: Yb, Er@NaYF_4_		PEI	Hydrophilia	NaYF_4_: Yb, Er@NaYF_4_-NH_2_/Calcium Red/Alizarin Red S	Sensing pH	[68]
NaYF_4_: Yb, Tm		Polyethyleneimine	Hydrophilia	NaYF_4_: Yb, Tm-NH_2_	Intracellular imaging	[69]
NaYF_4_: Yb, Er		APTES	Absorb negative charges	NaYF_4_: Yb, Er-NH_2_@SiO_2_-NH_2_ and AuNPs-citrate	Cyano-containing pesticides detecting	[70]
NaYF_4_: Yb, Er@NaYF_4_	COOH-	PAA in DEG	Hydrophilia	NaYF_4_: Yb, Er@NaYF_4_-COOH, OH	--	[71]
NaYF_4_: Yb, Tm		PAA in H_2_O	Covalent coupling of dopamine by amidization reaction	NaYF_4_: Yb, Tm-COOH-dopamine	Organophosphorus pesticide detecting	[72]
NaYF_4_: Yb, Er		PAA in ethyl alcohol	Coupling with hydrophilic materials	NaYF_4_: Yb, Er-COOH-RGB inks	Drug Anti-Counterfeiting	[73]
NaYF_4_: Yb, Er (Tm or Ho)		Lemieux-von Rudloff reagent (OA is oxidized)	Coupling with proteins	UCNPs-Strepta-vidin	DNA detecting	[22]
NaYF_4_: Yb, Tm		Methacrylic acid (MAA)	Loading CDDP	NaYF_4_: Yb, Tm-COOH	Drug delivery	[74]
NaYF_4_: Yb,Er		Adipic acid	Coupling the growth and hydrophilia	NaYF_4_: Yb, Er-COOH	Intracellular imaging in vitro	[75]
NaYF_4_: Yb, Tm	COOHCOOH	PAA in diethylene glycol	Coupling with antibody	NaYF_4_: Yb,Tm-COOH/Magnetic polystyrene microspheres	Bisphenol A detecting	[76]
NaYF_4_: Yb,Er	--	Poly-MAEP	Coupling with proteins	NaYF_4_: Yb, Er-MAEP	Cell imaging	[77]
NaLuF_4_: Yb, Er	--	Amphiphilic phospholipid functionalized poly ethylene glycol and DSPE-PEG	Amphipathy	NaLuF_4_: Yb, Er-ph-PEG, DSPE-PEG	Deep-tissue bioimaging	[78]
NaYF_4_: Yb, Er	Aryl group-	Phosphoryl-functionalized pillar arene	Hydrophilia	NaYF_4_: Yb, Er-PP5	pH-responsive DDS	[79]
NaYF_4_: Yb, Er	--	α-Cyclodextrin	Hydrophilia and specific recognize Cys	α-CD-NaYF_4_: Yb, Er-rhodamine-oxaldehyde (RHO)	Cys detecting	[80]
LaF_3_: Yb, Ho or LaF_3_: Yb, Er	--	Polyethylene glycol monomethyl ether	Amphipathy	LaF_3_: Yb, HO/LaF_3_: Yb, Er- mPEG-OH	The UCNPs epoxidation	[81]
NaYF_4_: Yb, Er	--	Polyethylene glycol-poly (lactic-co-glycolic acid) polymer,	Positive charge and amphiphilicity	NaYF_4_: Yb, Er- PEG-PLGA	Drug delivery	[82]
NaYF_4_: Yb, Er, Tm	Thiazole Derivative-	α-Cyclodextrin	Hydrophilia and specific recognize Hg^2+^	α-CD- NaYF_4_: Yb, Er, Tm	Hg^2+^ detecting	[83]

### 2.4. Mechanisms and Techniques for Optical Analysis of UCNPs Nano-Platforms in Practical Applications

Until now, The UCNPs emits high-energy visible light by two-photon or multi-photon processes under excitation with low-energy excitation light such as near infrared, which means that upconversion nanomaterials reduce background fluorescence and light scattering [84,85,86], thus allowing qualitative analysis with quantitative processes that have unpredictable effects and receive increasing attention from researchers [6]. In addition, the UCNPs with long fluorescence lifetime, high quantum yield, and low photodegradation have been widely used in therapeutic, environmental, biological, food, and medical applications [87,88].

UCNPs fluorescence probes combined with fluorescence analysis are often used in material analysis to use their luminescent properties. Fluorescent determination often utilizes fluorescence labeling or label-free fluorescence techniques [89,90]. The fluorescence labeling method presupposes labeling and separation of the sample from the target analyte, and the label-free fluorescence techniques are accomplished primarily by quenching, reducing, or restoring fluorescence affected by the analyte.

The fluorescence quenching (FQ) mechanisms are mainly as follows: (i) Static or dynamic quenching: weak binding between the ground state fluorescence molecule and quench agent produce new complex, which causes the static quenching; the excited fluorescent molecule collides with quench agent which causes the dynamic quenching. (ii) Energy transfer (ET): the two standard UCNPs fluorescence sensor energy transfer modes are FRET theory, and luminescence resonance energy transfer (LRET). These mechanisms describe ET from a donor fluorophore to an acceptor fluorophore through nonradiative dipole–dipole coupling. UCNPs are used as energy donors in ordinary, and it offers considerably greater freedom for upconverted emission wavelengths than the one produced merely by the lanthanide ions. Despite there being little difference between FRET and LRET, the energy transfer in LERT is radiative, whereas it is nonradiative for FRET, and the sensitivity and selectivity of the UCNPs fluorescent probe will not be influenced by this. (iii) The Inner filter effect (IFE) refers to the absorption of the exciting radiation and/or emitted fluorescence radiation of fluorophores by absorbers in the detection system. IFE only occurs effectively if the absorption band of absorbers possesses the complementary overlap with the excitation and/or emission bands of fluorophores. This theory depends on luminescent groups that can be applied in substance analysis [91]. Compared with those fluorescence probes that can be changed directly or indirectly through surface modification or functionalization, either independent transfer of the energy or the substance to be measured, the IFE-based approach does not require the link of absorbers with fluorophores, which offers considerable flexibility and more simplicity. (iv) Photoinduced electron transfer (PET) system when upon near-infrared excitation, the excited photoelectrons of UCNPs can be captured by electron acceptors. The maximal difference between PET and FRET is that two disparate fluorescent substances pass the energy in FRET, while PET only needs a vacancy in the fluorescent group to support electron transition, which can make the operation easier [92].

Amidst label-free fluorescence techniques, biological means purpose to FQ has been applied to analysis currently, except fluorescence quenching caused by physical factors such as immunofluorescence and nucleic acid aptamer unlabeled fluorescence. Indeed, the principles of these technologies are still inseparable from physical factors. The mechanism of label-free immunoassay is that the recognition process of antibody and antigen binds or separates with the fluorescent probe competitively, and then attenuation the fluorescence signal. Similarly, the unlabeled fluorescence detection mechanism of the aptamer is that the combination of the aptamer and the target changes the secondary structure of the aptamer. Furthermore, the competitive hybridization between the complementary sequence and the aptamer changes the environment around the aptamer and weakens the fluorescence signal of the fluorescent probe.

In addition to label-free fluorescence techniques used frequently for biological detection, the above-mentioned free-labeled fluorescence technique is usually characterized through fluorescence quenching. There are other kinds of analysis modes such as ratio fluorescent analysis and Colorimetric fluorescent detection [93]. In the upconversion fluorescent probe system, the solid electron-withdrawing group connected with the UCNPs and the strong electron-donating group or electron-donating organic dye connected with gold nanoparticles or quantum dots form a D-π-A conjugated system. During stimulation, donor-to-donor electron transfer is likely to occur, resulting in changes in fluorescence properties that can be implemented in the fluorescence signal transmission ratio test [94].

## 3. The UCNPs in Analytical Application of Environmental Science

The healthy water-cleaned soil and pure air in the natural environment are the cornerstone of the social environment, which is the human pursuit. Nevertheless, the progress of science and technology and the development of society also have related costs. There are heavy metal ions, organic pollutants, and waste residue in lakes and land; colored gas remains for a long time. Researchers have studied various effective methods for analyzing these ingredients, such as chromatography, which has high separation efficiency and poor qualitative ability. Moreover, mass spectrometry (MS) can detect organic compounds’ structural while because of complex samples to be tested, MS alone cannot meet the requirement of testing. The development of nanotechnology, due to the size of the nanoparticles’ advantages of luminescence properties, promotes the application of fluorescence detection, gradual development of nanometer fluorescent probes for analysis of toxic and harmful substances in the environment bring innovation. At the same time, compared with the expensive instrument analysis of the complex operation and strict sample pretreatment, nano fluorescent probe with simple operation and strong specificity supplies a mean for real-time detection.

The UCNPs as light-emitting tunable fluorescence sensors play a favorable role in monitoring pesticide residues, heavy metal ions, organic pollutants. Additionally, inorganic salt ions in water and soil, as well as harmful gases in the atmosphere [95,96,97]. Furthermore, we summarize the contribution of the UCNPs fluorescent nanoplatforms in environmental analysis in Table 3.

### 3.1. Organic Contaminant Residue Analysis

Common organic pollutants in the environment include organic pesticides, polycyclic aromatic hydrocarbons, dioxin compounds, polychlorinated biphenyls, phenolic compounds, etc. They are difficult to be degraded and can seriously endanger human life. Gas Chromatography-Mass Spectrometry and Liquid Chromatography-Mass Spectrometry are commonly used to detect these organic pollutants, which is more costly than the UCNPs fluorescence probe analysis we presented. Among them, organic pesticides such as organochlorine, organophosphorus, nitromethyl, and nicotine act on various crops by different toxic mechanisms. Take organophosphorus pesticides as an example, they inhibit acetylcholinesterase activity in the central and peripheral nervous system. While poisoning insects, their residues can affect human health. This chapter focuses on applying the UCNPs fluorescence analysis mainly in environmental wastewater, industrial effluents, soil contamination, etc.

**Table 3:** A summary of achievements in analysis of pollutants in environmental science through UCNPs platform.

Analyte	Matrix	Analysis Platform	Technique	Linear Detection Range (ng mL^−1^)	LOD (nmol L^−1^)	Recovery (%)	RSD (%)	Ref.
Metribuzin	Surface and ground waters	NaYF_4_: Yb, Er-Near Infrared dye	Ratiometric and colorimetric	4.93 × 10–3.21 × 10^2^	68	--	1	[98]
	Tap water, river water	NaYF_4_: Yb, Er-tetramethylrh odamine	Fluorescence turn on-off	0–80	2.19 × 10^−7^	91.0–115.0	2.3–3.7	[99]
Bisphenol A	Water sample	NaYF_4_: Yb, Er@Mn-aptamer	Electrogenerated chemiluminescence	0.05–100	1.62 × 10^−7^	98–102.50	--	[100]
	River water	NaYF_4_: Yb, Tm-MPMs ^1^	Immunofluorescence	1 × 10^5^–5 × 10^8^	8.76 × 10^−3^	85.35–108.35	--	[76]
Perfluorooctane sulfonate	Surface water	NaYF_4_: Yb, Er-BSTFA ^2^	Fluorescence quenching	1.5 × 10^3^–5 × 10^4^	2.43	85.8–118.6	9.8	[101]
Polychlorinated biphenyls	Water and soil samples	NaYF_4_: Yb, Er-BHQ ^3^-1, Carboxylated MMPs ^4^	Fluorescence turn on-off	0.004–800	1.36 × 10^−8^	93.4–109.7 83.2–118.5	1.6–2.9, 2.1–3.2	[102]
Ag^+^	Environmental water	NaYF_4_: Yb, Er/GQD ^5^	Fluorescence turn on-off	0.022 1–107.8	0.060	95–102	2.30–3.39	[103]
Cr^3+^	Industrial waste water	LiYF_4_: Yb, Ho@LiYF4@Ce^3+^	Ratiometric fluorescence	260–2600	4.1 × 10^2^	95.7–97.2	--	[104]
Fe^3+^	Waste water	NaYF_4_: Gd Yb, Ho/EPA ^6^	Ratiometric fluorescence	14–2800	2.5 × 10^2^	100.9–107.3	0.8–1.4	[105]
	--	LiYF_4_: Yb, Er, Ho, Tm@LiYF_4_: Yb	Fluorescence quenching	0–8.6 × 10^6^	--	--	--	[106]
Pb^2+^	--	NaYF_4_: Yb, Er@NaYF_4_/AuNPs	Fluorescence turn on-off	0–4.1	4.1	--	--	[107]
	Waste water	NaYF_4_: Gd, Yb, Ho/MNPs/AuNPs	Fluorescence turn on-off	2.05–114.8	5.7	99.6–105.2	0.9–2.2	[57]
Cu^2+^	Tap water	NaYF_4_: Yb, Er/ AuNPs-4-mercaptobenzoic acid	Fluorescence turn on-off	1.28–64	18.2	98–106	1.2–1.8	[108]
PO_4_^3−^	Aqueous samples	ZrO_2_: Yb, Er@ZrO_2_/Fast Green alimentary dye	Fluorescence turn on-off	1.9–95	20	--	--	[109]
SO_2_	SO_2_ gas in atmosphere	NaYF_4_@NaYF_4_: Yb, Tm/cyanine dye	Fluorescence turn on-off	1 × 10^−3^–1 × 10^3^	1.6 × 10^−2^	--	--	[110]

^1^ MPMs: magnetic polystyrene microspheres; ^2^ BSTFA: N, O-bis(trimethylsilyl) trifluoroacetamide; ^3^ BHQ: quenchers; ^4^ MMPs: magnetic microspheres; ^5^ GQDs: graphene quantum dots; ^6^ EPA: N, N-diethyl-p-phenylenediamine.

Organic herbicide processes forceful systemic, which can produce targeted toxicity. Although OCs have self-decomposition ability, their residual toxicity can last a long time. Sayed M. Saleh et al. [98] designed a ratiometric and colorimetric optical sensor film which consists of near-infrared (NIR) dye 2-[2-[2-Chloro-3-[2-[1, 3-dihydro-3, 3-dimethyl-1-(4-sulfobutyl)-2H-indol-2-ylidene]-ethylidene]-1-cyclopenten-1-yl]-ethenyl]-3, 3-dimethyl-1-(4-sulfobutyl) -3H-indolium hydroxide and UCNPs, making use of UCNPs can be emitted a dual (green and red) emission under 980 nm laser diode excitation. The NIR probe conjugated system has a chloro group to the nucleophilic substitution of amines which changing the electronic distribution of their conjugated system causes the difference fluorescence signal. Then, the metribuzin is to identify. This system is precise because polyvinyl chloride (PVC) polymer was utilized to provide high homogeneity protecting fluorescent probes from leaching out. The cocktail exhibits high stability over long periods of time, high reproducibility, and exposure to pesticide media exhibits high stability.

Bisphenol A, which is widely used in plastic products such as water bottles, is an environmental estrogen that can enter water or soil during plastic degradation, so bisphenol A (BPA) testing has become particularly important. Therefore, Li et al. [99] designed streptavidin and amino groups modified UCNPs, and the modification of amino groups can save the use of biological materials. Additionally, the detection limit can be as low as 0.05 ng mL^−1^. Compared to the above method, Guo et al. [100] took advantage of an aptasensor labeled with Mn^2+^-doped NaYF_4_: Yb, Er combined with electrogenerated chemiluminescence, which solved the oxidation of Bisphenol A near catechin potential sensitivity of analysis, which was improved. Fluorescence analysis of electroluminescence-assisted UCNPs improves the analysis speed and the detection limit is as low as 0.037 ng mL^−1^.

Many kinds of persistent organic pollutants are so due to their persistence, bioaccumulation, high toxicity, and complex environmental degradation, such as widespread contaminant PFOS. Li et al. [101] designed a fluorescence sensor, UCNPs@COFs, through hydrogen-bond interactions between COFS and PFOS to quench the UCNPs fluorescence. At the same time, COFS on the surface of UCNPs is ready to improve the fluorescence quantum yields and cause the sensor much more sensitivity. Tian et al. [111] prepared NaYF_4_: Yb, Er@NaGdF_4_@MIP to detect PFOS, and this fluorescent sensing can be used in a wide range of applications without affecting the upconversion of luminescence during specific identification.

**Figure 4:**
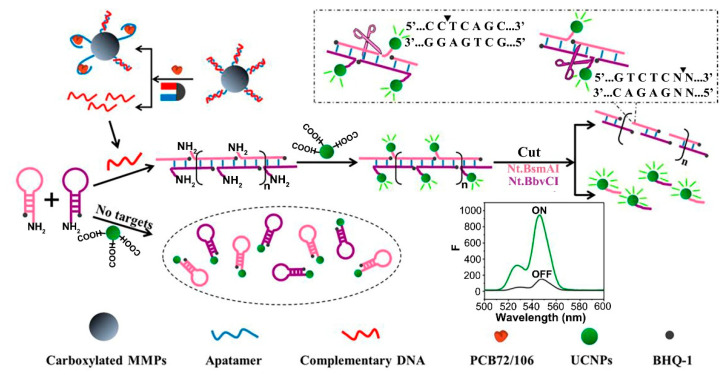
Schematic illustration of the dual-amplification strategy for PCB72/106 detection of upconversion fluorescent aptasensor based on HCR and nicking endonuclease. Reprinted with permission from [102]. Copyright 2018, American Chemical Society.

Wang et al. [102] prepared a UCNPs fluorescent aptasensor based on hybridization chain reaction and nicking endonuclease. This aptasensor has been developed to detect polychlorinated biphenyls. The robust aptasensor can be applied for the analysis of actual samples, and it also has high sensitivity and excellent selectivity (Figure 4).

The UCNPs have favorable biocompatibility, stably coupling with nuclein and amino acids to use a fluorescence immunoassay in organic pollutants analysis. Duan et al. [76] proposed a fluorescence immunoassay sensor to detect BPA with anti-BPA antibody conjugated carboxyl functionalized the UCNPs and coating antigen-conjugated carboxyl-functionalized magnetic polystyrene microspheres. This method can provide a suitable way for quick field analysis, which can be very accurate.

### 3.2. Inorganic Contaminant Analysis

In this section, we summarized recent works on the use of UCNPs for detecting various inorganic contaminants such as chemical ions. Moreover, the results also deliver real-time information on targeted ions in complex samples, and some gas pollutants.

#### 3.2.1. Heavy Metal Ion Analysis

Toxic heavy metals such as Chromium (Cr), Iron (Fe), Copper (Cu), Lead (Pb), Silver (Ag), and Mercury (Hg) are increasing discarded into environmental surroundings such as soil, river, lake, and pond water. What is more serious is that toxic heavy metals pose a threat to human existence indirectly. These metal ions can be analyzed explicitly in the light of luminous intensity changes in UCNPs, including luminescence enhancement, luminescence attenuation, and fluorescence quenching.

Among the rest, silver ions have excellent antibacterial activity, but a heavy metal contaminant can also cause irresistible side effects in humans. In 2017, He et al. [103], taking advantage of the ability of Ag^+^ to specifically bind to two cytosines (C) mismatch in DNA to form stable C-Ag^+^-C complexes, established Sodium citrate functional UCNPs and NH_2_-ssDNA functional graphene quantum dots. When adding Ag^+^ to this system affected by FRET, UCNPs are fluorescence quenching.

**Figure 5:**
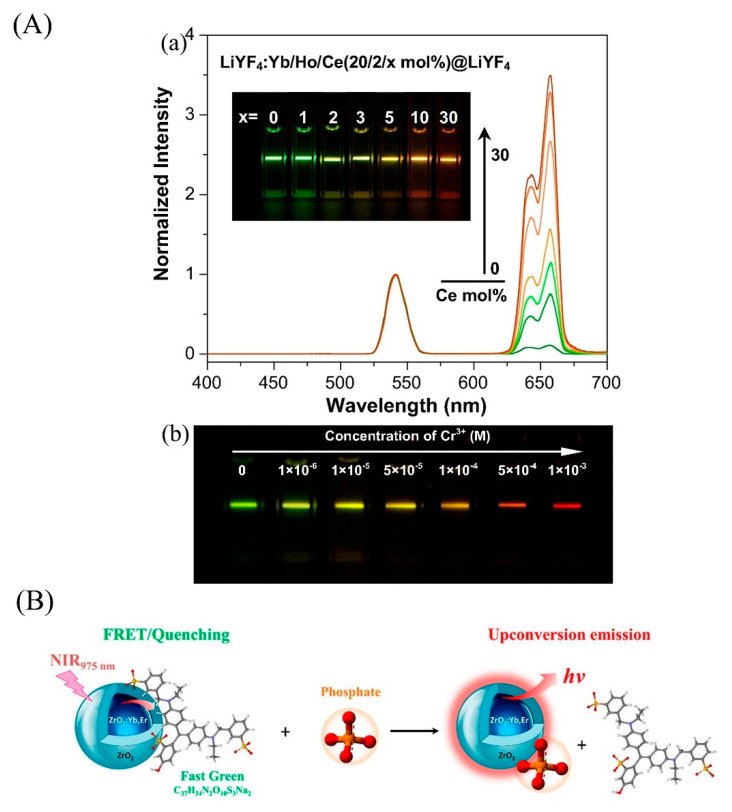
UCNPs for detecting heavy metal, inorganic acid ion and air pollutants. (**A**) (a) Upconversion emission spectra of the LiYF_4_: Yb^3+^/Ho^3+^/Ce^3+^(20/2/x mol%) @LiYF_4_ as a function of Ce^3+^ content in the core. The spectra were normalized by the Ho^3+^ emission intensity at 540 nm. The inset is the corresponding luminescence photographs. (b) Typical luminescence photos of the UCNPs-CRD probe solution under the excitation of 980 nm laser (15 W cm^−2^) at different concentrations of Cr^3+^. (**B**) Schematic illustration of the phosphate determination method based on FRET from ZrO_2_: Yb, Er@ZrO_2_ core@shell nanoparticles to the FG acceptor. Reprinted with permission from [104,109]. Copyright 2017, Elsevier and 2019, American Chemical Society.

Chromium is a trace metal element of the human body for physiological function, but excessive intake of chromium ions is seriously harmful to health. Liu et al. [104] used the luminance tunability of UCNPs to design a single structure Ce^3+^-doped LiYF_4_: Yb^3+^/Ho^3+^@LiYF_4_ modified by CRD through FRET. With the addition of Cr^3+^ into the probe solution, the color of the solution will be changed from green to other colors (Figure 5A).

#### 3.2.2. Inorganic Acid Ion

Phosphorus is required for plants growing. Ramirez-Garcia et al. [109] designed a nano-scale conjugated fluorescence sensor. The sensor uses conjugated luminescence of Fast Green alimentary dye (FG) and ZrO_2_: Yb, Er@ZrO_2_, as well as the strong interaction and spontaneous formation of chemical bonds between phosphate group and zirconia (ZrO_2_) as a phosphate monitor in the environment, as shown in Figure 5B. This sensor structure can enhance the upconversion luminescence, thus improving its sensitivity. While using FG, it is necessary to pay attention to its particular physical characteristics. It is therefore highly desirable to design better UCNPs-based nanosensors for PO_4_^3−^.

#### 3.2.3. Air Pollutants

Sulfur dioxide (SO_2_), nitric oxide (NO), sulfuretted hydrogen(H_2_S) as a dominant component of industrial waste gas. Additionally, it has become a key indicator in the monitoring of atmospheric contamination. Zhang et al. [110] set a paper-based sensor through cyanine modified UCNPs to detect SO_2_. On account of FRET between cyanine and UCNPs to decrease UCNPs luminescence, the absorption of the cyanine dye can be quenched after reacting with bisulfite ions, thereby increasing the luminescence of UCNPs.

It is sensitive to utilize test paper to monitor SO_2_ when building a smartphone-based detection platform, manufactured via 3D printing technology. It provides a highly feasible idea for the real-time monitoring of other gas pollutants such as NO or H_2_S.

## 4. UCNPs in Analytical Application of Bioscience

The UCNPs have shown significance as biomarkers compared to traditional fluorescent dyes because of unique anti-Stokes fluorescence properties [112]. The excitation of UCNPs bio-labels allows NIR light, it is non-invasive, and it can penetrate deep into cells and tissues. This property of the UCNPs make its fluorescence unaffected by the sampling fluid during the detection process. More importantly, NIR irradiation is not absorbed by biological samples and therefore does not produce an auto-fluorescent background [113]. Moreover, it significantly reduces the LOD that cannot be obtained by conventional assays. Therefore, the application of UCNPs in bioanalysis has obvious advantages.

### 4.1. Analysis of Biomacromolecule

Biological macromolecules, including proteins, enzymes, and nucleic acid, will reflect the body’s physiological state to a certain extent. Then, UCNPs, a slapping fluorescent probe, can monitor them and provide essential information for lesion diagnosis and later treatment.

#### 4.1.1. The UCNPs Analysis of Proteins

There are three kinds of commonly used analysis strategies for UCNPs to detect protein. (i) Constructing a FRET system with other factors; (ii) The UCNPs can be used as a medium to build a hairpin DNA probe, and the premise is that the probe contains a substance which can be identified explicitly with the protein being tested. (iii) UCNPs use identified protein antibodies as vectors. Protein detection can be realized using the advantage of UCNPs biological binding, combining instruments, or constructing fluorescent side-flow biosensors.

Heparin is a polysaccharide that works as a clinical anticoagulant drug to prevent blood clotting, while heparin overdose and prolonged use could induce potentially fatal bleeding complications. Additionally, protamine is the antidote for heparin. Long et al. [114] designed the UCNPs-AuNPs FRET system formed by the electrostatic adsorption, which will quench UCNPs, because the protamine has much positive charge and it can bind to AuNPs to make UCNPs fluorescence recovery. Additionally, because heparin is electron rich, adding it to this solution will combine with protamine, and then UCNPs fluorescence will be quenched again. The sensitivity of this dual function probe is relatively high, and the detection line of heparin is decreased to 0.7 ng mL^−1^.

Alpha fetoprotein (AFP) is an important cancer biomarker of liver cancer, currently detecting AFP relays via instrumental means. Hu et al. [115] established an immunoassay based on ICP-MS using UCNPs as elemental tags to determine AFP. The proposed method is rapid and accurate. Moreover, it has good tolerance to complex biological matrices, indicating the great potential of UCNP as an elemental marker in biological research. Zhai et al. [116] prepared NaYF_4_: Yb, Tm/ZnO/CdS composite film electrode by pulsed laser deposition, prompting the photoelectrochemical performance of the electrode, and more importantly it shows ultrasensitive detection of AFP. Its low detection limit can reach 5 pg mL^−1^. It is one of the best results of all AFP analyses. A similar consequence can be achieved through the photoelectrochemical (PEC) immunoassay sensing technique. Luo et al. [117] used the high catalytic activity of glucose oxidase (GOx) to construct core–core–shell UCNP@Au@CdS and generated H_2_O_2_ using polyclonal anti-AFP antibodies. A sandwiched immunoreaction was first performed in microtiter plates coated with monoclonal anti-AFP antibodies. Additionally, antibody-labeled gold nanoparticles were utilized as secondary antibodies to promote photocurrent signals.

While one limitation of this method is that the generated H_2_O_2_ by enzyme immunoassay is artificially injected into the photocurrent detection cell, we need to simplify the process for experimental purposes.

Target-triggered DNA assembling probes labeled with UCNPs mediated amplification strategy provide more affinity sites to improve system sensitivity. Liu et al. [118] designed a target-triggered DNA assembling probe for a specific analysis of growth factor-BB. Among them, the hairpin DNA (H-DNA) probe was designed containing (a) an aptamer domain for protein recognition and (b) a blocked DNAzyme domain for DNA-zyme cleavage. Once H-DNA and A-DNA recognize the same protein, H-DNA and A-DNA are near each other. The unfolded DNAzyme hybridized with the added MB-UCNP and catalyzed the cleavage of the MB-UCNP amplification signal when Zn^2+^ is added as a cofactor.

The UCNPs probe includes two affinity sites. The target proteins binding to two affinity probes can be applied in this strategy. Such UCNPs probes can only detect specific proteins because of the limitation of relying on one or more affinity sites. If there are no affinity sites in the protein, we cannot use this method. At the same time, it is not such a suitable means for most proteins, and it will take too much time and is much expensive. A sensitive and straightforward UCNPs probe to detect growth factor-BB (PDGF-BB) will be designed, just like a direction of effort.

Some enzymes as specific proteins in organisms have a heavy response with the UCNPs fluorescent platforms, which can shred evidence to inform clinical diagnosis. Alkaline phosphatase (ALP) is a necessary serum biomarker that is an essential indicator of clinical diagnostics. Chen et al. [119] designed an enzyme cascade signal-amplified (ECSAm) strategy with label-free silver triangular nanoplates (AgTN-Ps) combined with UCNPs that can realize rapid and accurate recognition of ALP in serum, while this process is not as complex when compared with a “turn-on” sensor based on MnO_2_-coated UCNPs prepared by Liang et al. [120]. Because only one reaction of ascorbic acid and MnO_2_ reduction is introduced, it simplifies the operation of the experiment. The weakness of this method is that its LOD compared with the former 0.035 mU mL^−1^ is a little higher.

Bifunctional UCNPs can detect metal ions simultaneously while detecting enzymes, due to the ability of some metal ions to form complexes with enzymes, such as (GSH)_4_Cd complex, which is formed by the aggregation of Cd^2+^ and GSH, and Fang et al. The authors of [121] used GSH as a link to detect acetylcholinesterase (AChE) through AuNPs-UCNPs FRET sensing platform. Moreover, the detection limit of AChE activity is 0.015 mU mL^−1^.

#### 4.1.2. The UCNPs Analysis of Nucleic Acids

The aberrant expression of MicroRNAs (miRNA) is usually associated with human cancer. The diagnosis and discovery of miRNA at an early stage of the disease are essential. The abundance of miRNA is a little low, and PCR can amplify mRNA detection accuracy in common, while the fee is costly. Researchers use chemical methods by introducing UCNPs, which have no background autofluorescence, no photobleaching, and stable luminescence, and which detect miRNA in the cell.

In order to achieve rapid, simple, and sensitive analysis of miRNA, many researchers have designed the contact sites and structural regulation of UCNPs (Table 4).

Because RNA sequence fragment signals are pretty weak, sequence amplification is necessary, but it is complex. Many researchers are devoted to combining UCNPs FL sensors with DNA or produce devices, because it can amplify signals to achieve sensitive and speedy analysis of mRNA without amplification. This design brings many challenges to the experiment and becomes a problem to be overcome in future research.

### 4.2. Analysis of Small Biomolecules

Biological small molecules including amino acid and polypeptide et al. For example, the chemical structure of amino acids is easily captured by substances modified on the surface of UCNPs. Or it can be directly designed as a fluorescence resonance energy transfer IFE sensor. This has also become one of the ordinary means to detect other small biological molecules on the UCNPs nano platform.

#### 4.2.1. The UCNPs Analysis of Amino Acid

Associated with detecting arginine, Wu et al. [122] used the guanine group in the positively charged arginine to bind to the AuNPs electrostatically to designed a FRET system between the UCNPs and AuNPs, and the limit of detection is as low as 2.9 μmol L^−1^. Using UCNPs to analyze tyrosinase can show a dual-functional sensor, as Wang et al. [123] studied, since the tyramine under the action of tyrosinase generated melanin polymers can convert fluorescence quenching. Additionally, the fluorescence intensity has a linear relation with the concentration of tyramine. Moreover, the result has a linear relation and the activity of tyrosinase, to realize the double function test. The content of tyrosinase was measured indirectly, and the analysis of tyrosinase detection limit was as low as 0.003 U mL^−1^.

#### 4.2.2. The UCNPs Analysis of Peptides

Glutathione can reflect the human immune system in some conditions, and some researchers are interested in detecting it. Sun et al. [53] designed bifunctional UCNPs fluorescence probes to detect glutathione (GSH) and Cd^2+^, and the probes have consisted of UCNPs and AuNPs. Compared with Nguyen et al. [124] utilizing Rhodamine B derivative UCNPs to detect GSH by the FRET principle, the last method is a little expensive, and this way provides a much easier choice, with no background fluorescence. Moreover, Chen et al. [125] built core–satellite UCNPs and introduced CaF_2_, thus enhancing the absorbability of biomass. The key to this method is constructing the UCNPs structure by the sequential injection technique. Most importantly, it can produce specific recognition of GSH.

#### 4.2.3. The UCNPs Analysis of Neurotransmitter

**Figure 6:**
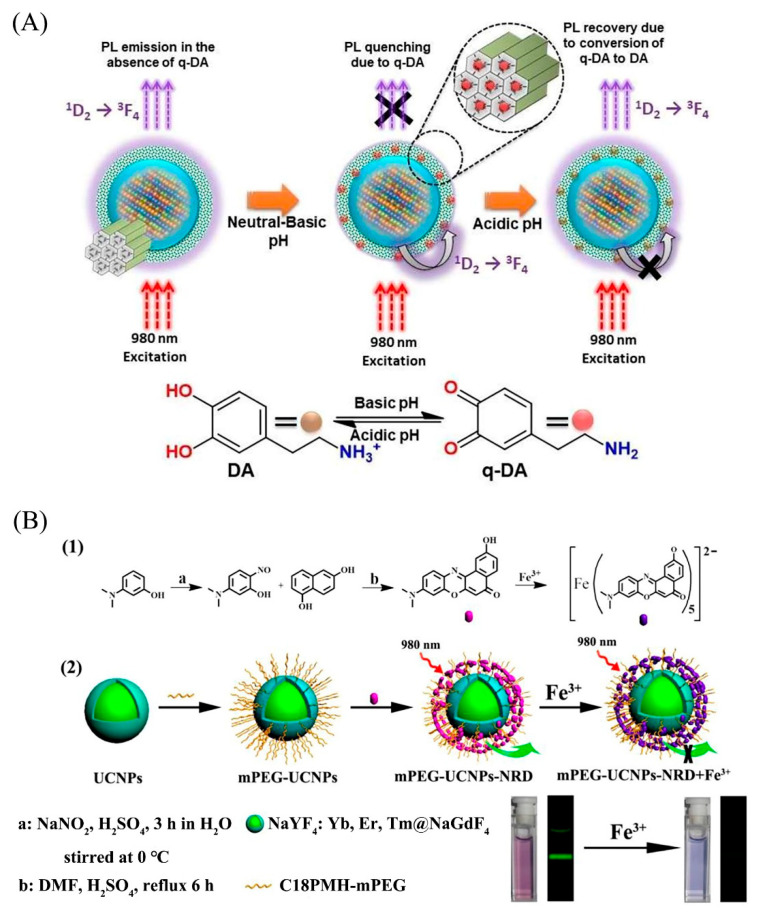
UCNPs in Analytical Application of Bioscience. (**A**) Concept of upconversion based nanoplatform (BCSU-MS) for dopamine (DA) and pH sensing. (**B**) (1) Illustration of the Synthesis of Nile Red Derivavite (NRD), (2) mPEG-UCNPs-NRD and Their Use for Detecting Fe^3+^ Based on Change in UCL Emission. Reprinted with permission from [127,153]. Copyright 2019, John Wiley and Sons and 2016, American Chemical Society.

Dopamine (DA) is the most abundant catecholamine neurotransmitter in the brain. In contrast, clinical diagnosis of dopamine is often unsuitable for routine analysis, due to the high cost, long analysis time, complex pretreatment, and low selectivity to dopamine in the analysis process. Jose et al. [126] used UCNPs as a resonant light scattering (RLS) nanometer probe for quantitative analysis of dopamine, achieving trace analysis of biological samples with accurate detection data and excellent selectivity. Furthermore, Kumar et al. [127] developed a facile UCNPs sensing platform to detect DA in real time, wherein the LOD is as low as 0.63 nmol L^−1^ and the method is highly sensitive and agile (Figure 6A). Early sensitivity analyzation of thyroid-stimulating hormone (TSH) allows for early diagnosis of thyroid related disorders. Liu et al. [128] designed the UCNPs-tetramethylrhodamine (TAMRA) graft aptamers for the detection of TSH. When TSH bound to the aptamer to form a stable hairpin structure, the distance between UCNPs and TAMRA was reduced, thus causing the fluorescence quenching of NaYF_4_: Yb, Er, so that the TSH can be detected rapidly in serum.

### 4.3. Analysis of Microorganism

It detects microorganisms such as fungi, mycotic, pathogens, viruses, etc., that utilize electrostatic interactions between UCNPs and microorganisms and unique fluorescence properties of UCNPs. This chapter’s content lies in elaborating the application of upconversion fluorescence analysis in the biological context. However, the collation found the detection of microorganisms in this section, and it has a pattern with the analysis of microorganisms in the food context. Then, in this section, a complete description of the UCNPs is applied to the analysis and detection of microorganisms. Chapter 5 will not be repeated. The specific content is organized as follows.

There are five patterns of microbial detection: (i) the UCNPs modified with antibodies to capture antigens assembled by magnetic nanoparticles (MNPs) or other analogs and utilizing the luminescence properties of UCNPs; (ii) the energy donor is composed of UCNPs attaching to antibodies or aptamers, and the energy receptor consisted of AuNPs or other nanoparticles or fluorescent quenchants combined with antigens or cDNA; (iii) the UCNPs as a carrier were used on the electrochemical immune analysis, and the lateral flow immune paper-based for detection is derived, which is based on the immune analysis; (iv) utilizing UCNPs luminescent signal can be adjustable to detect microbes through grafting optical fiber material or mesoporous-doped material; (v) combining with double or more modes for rapid real-time detection.

Here, the list is as follows, according to the selection of fungal toxin to be tested (Table 5).

**Table 4:** Summary of experimental results for miRNA detection.

Detector	Fluorescence Probe	Detection Method	Highlight	LOD	Linear Range	Ref.
mRNA	NaGdF_4_: Yb^3+^, Er^3+^/AuNPs@Pt satellite assemblies	Fluorescence analysis	In situ imaging and quantification of TK1 mRNA in live cells.	0.67 fmol (10 μg RNA) ^−1^	1.17–65.21 fmol (10 μg RNA) ^−1^	[129]
miRNA-21	NaYF_4_: Yb^3+^, Er^3+^-DNA H_1_	Inductively coupled plasma-mass spectrometry	Sensitivity.	0.041 fmol L^−1^	0.1–500 fmol L^−1^	[130]
miRNAs	NaYF_4_: Yb^3+^, Er^3+^-NH_2_/NaYF_4_: Yb^3+^, Er^3+^-COOH/ dye UC	Fluorescence analysis	--	5 × 10^5^ fmol L^−1^	2 × 10^5^–1.4 × 10^6^ fmol L^−1^	[131]
miRNA-155	NaGdF_4_: Yb^3+^, Er^3+^@NaYF_4_-DNA/AuNPs	Fluorescence analysis	--	4.5 × 10^3^ fmol L^−1^	0.1 × 10^5^–1.5 × 10^6^ fmol L^−1^	[33]
miRNA-21, miRNA-10b	NaYF_4_: Yb^3+^, Tm^3+^, Er^3+^-Ti_3_C_2_	Fluorescence analysis	Assisted single-stranded replacement double-amplified RNA detection of mRNA in cell lysates without complex equipment, allowing detection of different sequences of RNA according to test requirements.	--	5–1 × 10^5^ fmol L^−1^	[132]
miRNAs	CaF_2_: Yb^3+^, Ho^3+^@MSNs@SiO_2_-ssDNA/Polyurethane fibers@GO	Fluorescence analysis	Enrichment of RNA and greatly improves accuracy.	2 × 10^4^ fmol L^−1^	--	[133]
miRNA-21	Fe_x_Cu_y_Se@NaYF_4_: Yb^3+^, Tm^3+^	Fluorescence analysis and magnetic resonance imaging	Dual signals for in situ quantitative imaging analysis.	0.0058 amol (ng RNA)^−1^	0.035–31.824 amol (ng RNA)^−1^	[134]

**Table 5:** Microorganisms analyzed by the UCNPs nanoplatform in the last five years.

Microorganisms/Analytes	Fluorescence Probe	Detection Mechanism	Detection Method	Linear Range (ng ml^−1^)	LOD (ng ml^−1^)	Sample	Ref.
Dipi-colinic acid	NaYF_4_: Yb, Er-TPP ^1^/EBT ^2^	Luminescence	Colorimetric assay protocol	334–3.34 × 10^4^	1.5 × 10^2^	Human serum	[135]
Deoxynivalenol	Antibody-NaYF_4_: Yb, Tm, Gd/Antigen-MNPs	Luminescence	Immunofluorescence analysis	0.001–0.1	0.001	Adulterated oil	[136]
E. coli.	cDNA-NaYF_4_: Yb, Er/ Aptamers-AuNPs	FRET	Fluorescence analysis	5–106 (cfu mL^−1^)	3 (cfu mL^−1^)	Tap/pond water and milk	[137]
	cDNA-NaY/GdF_4_: Yb, Ho/Aptamer-MNPs	Luminescence	Immunofluorescence analysis	58–58 × 10^6^ (cfu mL^−1^)	10 (cfu mL^−1^)	Adulterated pork	[138]
	Aptamer-NaYF_4_: Yb, Er@NaYF_4_/WS_2_^3^	FRET	Fluorescence analysis	85–85 × 10^7^ (cfu mL^−1^)	17(cfu mL^−1^)	Tap water, green tea powder	[139]
Fumonisin B1	cDNA-NaGdF_4_: Yb, Er/AuNPs	FRET	Fluorescence analysis	1 × 10^−5^–0.1	3 × 10^−6^	Corn	[140]
Microcystin-LR	NaYF_4_: Yb, Tm@NaYF_4_: Yb-COOH/MoS_2_	Luminescence	Fluorescence analysis	0.01–50	0.002	Tap water	[141]
Mycotoxins zearalenone	cDNA-NaGdF_4_: Yb, Er/AuNPs	FRET	Fluorescence analysis	0.05–100	0.01	Corb	[140]
Ochratoxin A	NaYF_4_: Yb, Er/GO ^4^	FRET	Fluorescence analysis	0.001–250	0.001	Beer	[59]
	NaYF_4_: Yb, Er@Mn/Fe_3_O_4_NPs	Luminescence	Indirect competitive immunofluorescence analysis	--	9.553 × 10^−3^	--	[142]
	Aptmer-NaYF_4_: Yb, Er	Luminescence	Immunofluorescence analysis	5–100	1.86	Spiked wheat and beer	[143]
	NaYF_4_: Yb, Er/AuNPs	FRET	Fluorescence analysis	0.1–1000	0.022	--	[144]
	NaYF_4_: Yb, Tm/Polystyrene beads	Luminescence	Indirect competitive immunofluorescence analysis	--	0.34721	--	[145]
Polymyxin B and polymyxin B-resistant bacteria	NaGdF_4_: Yb, Er/AuNPs	Luminescence	Fluorescence analysis	--	--	Clinic urine	[146]
Single Escherichia coli	KLu_2_F_7_: Yb, Er/Tapered optical fiber	Luminescence	Fluorescence analysis	--	--	--	[147]
Staphylococcal Enterotoxin B	NaGdF_4_: Yb, Er/AuNR@Pt	FRET	Fluorescence analysis	2 × 10^−3^–0.4	0.9 × 10^−3^	Spiked milk	[148]
	NaGdF_4_: Yb, Er/AuNPs	Luminescence	Simultaneous detection of surface-enhanced Raman, fluorescence and circular dichroism modes	1 × 10^−3^–0.75	--	Spiked milk	[149]
T-2 Toxin	NH_2_-NaYF_4_: Yb, Er@SiO_2_/Fe_3_O_4_ MNPs ^5^	Luminescence	Fluorescence analysis	0.1–100	0.035	Beer	[140]
Yersinia pestis EV76	NaYF_4_: Yb, Er	ECL ^6^ signal	Electrochemical immunoassay	--	1.2 × 10^4^ (cfu∙mL^−1^)	Soil	[150]
Listeria monocytogenes	NaGdF_4_: Yb, Er/MNPs	Luminescence	Fluorescence analysis	68–68 × 10^6^ (cfu mL^−1^)	8 (cfu mL^−1^)	Pasteurized milk	[151]
Staphylococcus aureus	NaYF_4_: Yb, Er/AuNPs	FRET	Fluorescence analysis	47–4.7 × 10^7^ (cfu mL^−1^)	10.7 (cfu mL^−1^)	Pork, beef	[152]

^1^ TPP: sodium tripolyphosphate; ^2^ EBT: eriochrome black T; ^3^ WS_2_: layered tungsten disulfide; ^4^ GO: graphene oxide; ^5^ MNPs: magnetic nanoparticles; ^6^ ECL: electrochemiluminescence.

### 4.4. Analysis of Inorganic Substances in Biological Samples

According to the current researchers’ statistics of the research results, the UCNPs for the analysis of inorganic substances applied to biological materials can be divided into the following three categories: inorganic ions, reactive oxygen species, and gas molecules.

#### 4.4.1. The UCNPs Analysis of Inorganic Ions in Biological Samples

Inorganic ions play a critical role in the process of life. Wei et al. [153] designed an ingenious FRET system in which Fe^3+^-responsive Nile red derivative (NRD) was used as an energy donor, and the PEGylated amphiphilic polymer-modified UCNPs was used as energy acceptor. In addition, most of the Fe^3+^ selective probes are insoluble in water, so this structure solved this problem, and it is beneficial to the detection of Fe^3+^ in vivo, and its application in imaging has been proved (Figure 6B). Zhao et al. [154] utilized the Fenton reaction, the hydroxyl group produced when Fe^2+^ and hydrogen peroxide exists in vivo reacts with IR-808, and then the fluorescence resonance particles were increased to quantify Fe^2+^ accurately. Jiang et al. [155] improved the method of detecting Fe^2+^ in serum by designing a fluorescence sensor based on FRET between NaYF_4_: Yb, Tm and MnO_2_. After the addition of Fe^2+^, MnO_2_ was reduced to Mn^2+^, thus restoring the fluorescence of UCNPs. Mercury ion is a potent neurotoxin that accumulates in human bodies and causes severe nervous system damage.

Then, Huang et al. [156] utilized T-Hg^2+^-T base pairs to stabilize the two thymine (T) mismatched DNA to design DNA-functionalized upconversion nanoparticles. After adding Hg^2+^, the fluorescence of DNA-UCNPs will be quenched.

Compared to these two methods, the structure of the former fluorescence probe is simpler and easier to operate, while the latter is significantly more complex. However, the latter has a broader range of applications. For example, it can be applied to the environment in tap water or human urine, while the former is used for cellular environmental analysis. Additionally, readers can choose appropriately according to their needs. The detection range of the former (0–8 μmol L^−1^) is not as wide as that of the latter (10–10^4^ nmol L^−1^).

Potassium ions are electrolytes essential for homeostasis in the body. Real-time tracking of potassium concentrations in body fluids can provide important information for biomedical diagnosis, assessment of therapeutic interventions and optimization of exercise performance [157,158]. Chen et al. combined NaYF_4_: Yb, Tm (U-Tm, blue emission) and NaYF_4_: Yb, Er (U-Er, green emission) UCNPs with AuNP quenchers to construct two DNA-assembled nanosensors for monitoring H^+^ and K^+^ in the lumen of lysosomes. The different DNA-based sensor is capable of imaging both H^+^ and K^+^. The sensor can correlate the K^+^ concentration and pH in the lumen of the lysosomes, probing during lysosomal acidification to answer important biochemical and cell biological questions [159].

#### 4.4.2. Upconversion Analysis of Reactive Oxygen Species

Reactive oxygen species (ROS) produced through the body’s metabolism, including superoxide (O_2_^−^), hydrogen peroxide (H_2_O_2_), hypochlorite (ClO^−^), etc., play critical roles in many physiological processes.

Hao et al. [160] assembled a structure that analyzed ROS extensively, consisting of a UCNP core and a zeolitic imidazolate framework-8 (ZIF) shell encapsulated with chiral NiSx NPs. In vivo, H_2_O_2_ was successfully monitored by proportional assembly signal to verify its potential. The emission peaks of UCNPs doped with different rare-earth ions are different, and this property was to design the ratio fluorescent probes. Cy808 dye was used to enhance the luminescence of UCNPs by Zou et al. [161], and the excitation light of 808 nanometers was reduced while the excitation light of 980 nm remained unchanged after the addition of ClO^−^, to realize the identification of ClO^−^, which was successfully applied in mice with high sensitivity and a detection limit of 3.6 nmol L^−1^.

H_2_O_2_ is one of the critical ROS molecules, which can provide very favorable evidence for significant diseases such as malignant tumor detection in vivo. Chen et al. [162] used an enzymatically controlled amplification strategy of circulating signals and designs colorimeter chains with acetylcholinesterase involved in forming H_2_O_2_ and Fenton reactions to detect H_2_O_2_ and glucose lactic and uric acid. This process can detect a variety of small molecules in vivo while analyzing and detecting H_2_O_2_, but the whole process for detecting H_2_O_2_ is a little complicated. Singh et al. [163] synthesized an UCNPs embedded organometallic complex HNPs, NaYF_4_: Tm^3+^/Yb^3+^-Eu (TTA)_3_Phen (ETP), which has great sensitivity to H_2_O_2_ and can be used many times without losing sensitivity. However, this framework may be more toxic to organisms. Wang et al. [164] designed a FRET system consists of an energy donor, a novel Nd^3+^-sensitized UCNPs and the energy receptor dicyanomethylene-4H-pyran (DCM)-H_2_O_2_; utilizing the ratiometric fluorescence probe, the UCNPs luminescence (540 nm/660 nm) signal could visualize the H_2_O_2_ level. Additionally, the LOD was quantified to be 0.168 μmol L^−1^. This structure cannot cause an internal heat effect.

#### 4.4.3. The UCNPs Analysis of Hydrogen Sulfide in Biological Samples

Hydrogen sulfide (H_2_S) is a gas signaling molecule used to monitor acute pancreatitis in organisms and colorectal cancer [165]. Chen et al. [166] designed a sensing platform consisting of NaGdF_4_: Yb, Er@NaGdF_4_: Yb, Nd and IR-783. The dye-sensitized UCNPs nanoprobe has a dual-function of enhancing upconversion luminescence efficiency and specific recognition of H_2_S, and the detection limit is as low as 34.17 nmol L^−1^. Wang et al. [167] proposed poly (acrylic acid)-modified UCNPs assembled with cationic near-infrared cyanine chromophores (Cy_7_-Cl) to detect H_2_S in living cells and zebrafish, which can increase fluorescence of Cy_7_-UCNPs, and completed the imaging work in vivo. Liu et al. [168] took Prussian Blue (PB) as the responder of H_2_S, adding UCNPs performed by 5-nanometer lead envelopes. Employing the thickness of the lead envelope to detect H_2_S in the serum range, the deficiency of Wang et al. [167] organic dye with specific toxicity was improved during imaging and detection. It can not only possess remarkable H_2_S detection capacity, the LOD is 50 nmol L^−1^, but was also feasible for H_2_S elimination by UC-PB, reducing acute pancreatitis-associated lung injury and thus having excellent therapeutic potential as a drug.

### 4.5. Others

Glucose is the energy source of living cells and the intermediate product of metabolism in the body, and the content of glucose in the body is the data source for the diagnosis of diabetes. This is because glucose oxidase catalyzes glucose to produce gluconate hydrogen peroxide, which is associated with hydrogen peroxide usually during glucose analysis. Chen et al. [162] made use of this principle coupled with hydrogen peroxide, which catalyzes the conversion of ferrous to trivalent iron, and the trivalent ferric acid complex covers the converted fluorescence to obtain the analysis of glucose content. The experimental results are available, and the LOD can reach 2.3 μmol L^−1^. UCNPs can superiorly reflect the advantage of removing background fluorescence in detecting tiny molecule organisms such as urea. Long et al. [91] utilized IFE effective between oxidizing o-phenylenediamine and NaYF_4_: Yb^3+^, Tm^3+^, the low detection limit of uric acid is 6.7 μmol L^−1^. While this complex procedure contains two oxidation processes, Zhou et al. [169] simplified the experiment by the process of adding Fe^2+^ to the hydroxyl radical reaction product of the oxidation reaction of uric acid. Furthermore, the detection limit is also much lower than before, as low as 1.90 × 10^−3^ μmol L^−1^.

The mutagenic effects of drug residues on the hormonal action of organisms and bacterial resistance pose a continuous threat to the health of organisms. Furthermore, it has been a matter of immediate concern to medical experts in recent years. The determination of content of synthetic drug molecules in biological fluids has great significance in analytical biochemistry, clinical medical diagnosis, and local analysis or monitoring. The use of UCNPs for synthetic drug molecules sensing has attracted extensive interest because of their outstanding properties. The fluorescence of lanthanum (III) complex, produced by LRET from core–shell UCNPs, is sensitive to drug molecules. Hu et al. [58] utilized Yb^3+^-Yb^3+^ energy migration was doping in NaGdF_4_: Yb^3+^, Er^3+^@NaYF_4_: Yb^3+^ can enhance luminescence intensity, when adding doxorubicin to the solution, doxorubicin can quench UCNPs by LRET. Additionally, the doxorubicin concentration of 0.005 µg g^−1^ in the mice can be successfully detected through the upconversion fluorescent probe. According to a similar principle, Mo et al. [170] prepared NaYF_4_: Yb^3+^, Er^3+^, Nd^3+^@NaYF_4_: Nd^3+^ and successfully detected epirubicin, the LOD is 0.05 µmol L^−1^.

Alprenolol is a drug for hypertension, while on the other hand, it has been designated a forbidden drug by the International Olympic Committee, because of its improper use related to controlling physical activity to minimize the heart rate, coronary blood flow, etc. Thus, Lee et al. [171] designed amphiphilic functional UCNPs which are ultra-highly selective for alprenolol. The electrostatic interaction of conjugated polythiophene functionalized NaLuF_4_: Yb, Er with alprenolol resulted in specific recognition in human urine and serum with detection limits as low as 0.22 nmol L^−1^.

The fluorescence properties, nice biocompatibility, and low background fluorescence of UCNPs can be used to monitor nutrients in crops and provide reference information for economic decisions of nutrient management. Guist et al. [172] used UCNPs (NaYF_4_: Er^3+^, Yb^3+^) and Graphene Oxide to detect Zn deficiency in crops. At the time, smartphone color recognition had been successfully applied in this technology, which can quickly analyze Zn deficiency in barley or other crops. While the presence of a certain amount of UCNPs in organisms is also associated with specific toxicity, Modlitbová et al. [173] studied radish (Raphanus sativus L.) and common duckweed (Lemna minor L.) as adsorbed water-soluble UCNPs, as well as the toxicity, and bioaccumulation in these plants of UCNPs. Additionally, the length of the root per plant and the length of the hypocotyl per plant after 72 h exposure were chosen as toxicity endpoints, whereby 100 mg mL^−1^ of UCNPs/SiO_2_-COOH was highly toxic.

On the other side, Popov, etc. [174] used fluorescence signals of the UCNPs in zebrafish and shrimps to quantitatively evaluate the temperature and pH of aquatic organisms’ living environment in real time to determine the change in stress felt by organisms with the change in environment.

## 5. UCNPs in Analytical Application of Food and Medical Science

### 5.1. Analysis of Food Samples

Analysis of food sensing applications using UCNPs probes depends on several factors, including fluorescence, detection platform, and detection method. To achieve a specific and selective detection, UCNPs are functionalized with biological and chemical elements for target recognitions via typical antigen–antibody pairing, hybridization between complementary base pairs of nucleic acid, ionic interactions, etc. This chapter introduces the application of UCNPs in food analysis (milk, eggs, fruits, meat, drinking water), including residues of toxic and harmful substances, qualitative and quantitative analysis of harmful substances in food for humans, and the analytical application of enzymes and other active ingredients and summarizes the analytical profits in Table 6.

Food safety has always been an important issue related to human life safety, and the detection results of toxic and harmful substances residues in the diet are always an essential basis for food safety. After many experimental analyses on converting organophosphorus pesticides, nicotine pesticides, and other analytical applications, many researchers have obtained many research results. Organophosphorus pesticides (OPs) are widely used in agriculture because of their low persistence under natural conditions, easy synthesis, low cost, and high effectiveness for insect eradication. At the same time, they are neurotoxic due to their inhibition of acetylcholinesterase (AChE) in the central and peripheral nervous systems. Glyphosate is one of the OPs, Liu et al. [90] developed a colorimetric and fluorometric method based on a system composed of poly-ethylenimine-capped NaGdF_4_: Yb, Er, copper (II) ions, hydrogen peroxide, and 3,3′,5,5′-tetramethylbenzidine. The UCNPs fluorescence of UCNPs can be quenched owing to the strong coordination between poly-ethylenimine and Cu (II) with the presence of glyphosate. So, it has good specificity. Fenitrothion [O, O-dimethyl O-(4-nitro-m-tolyl) phosphorothioate is also a broad-spectrum organophosphorus insecticide. The study of Yu et al. [175] was based on one immunochromatographic strip to detect 2,4-dichlorophenxyacetic acid and fenitrothion, utilizing specific recognition of anti-2,4-dichilorophenoxyactice acid immunoglobulin G (2,4-D-IgG) and rabbit anti-fenitrothion IgG combined with UCNPs medium to implement fluorescence detection. This method can realize real-time detection, which is easy to operate, while the preparation of rabbit antibodies remains to be commercialized.

**Table 6:** Application of UCNPs platform in food science.

Target		Samples	Platform	Characteristic	Linear Detection Range (ng mL^−1^)	LOD (ng mL^−1^)	Recovery (%)	RSD (%) (n=3)	Ref.
Organophosphorus pesticides	Chlorpyrifos	Apples, cucumbers	NaYF_4_: Yb, Er-ChOx-Fe^2+^	Accurate identification of chlorpyrifos through double-enzymes	20–2000	6.7	89.5–97.1%	--	[176]
	Malathion	Tap water, matcha	NaYF_4_: Yb, Er-AuNPs	PDDA aptamer can specifically recognize malathion	3.3036–330.36	0.47	99–105.25 90–111.75	--	[177]
	Parathionmet-hyl, monocrotophos, dimethoate	Apples, cucumber, capsicum	NaYF_4_: Yb, Er–AuNPs–AChE-acetylthiocholine (ATC)	Ensure AuNPs does not aggregate in the presence of pesticides and resulting in high efficiency of FRET	0.002–0.2	6.7 × 10^−4^ 0.023 0.067	96.67–110.00	4.78–8.43	[178]
	Glyphosate	Instant tea	NaGdF_4_: Yb, Er-Cu^2+^-H_2_O_2_-TMB	Fluorescence of NaGdF_4_: Yb, Er at 660 nm increases linearly to form colorimetric assay	250–1.25 × 10^5^	9.8	96.4–100.74	0.56–2.91	[90]
	Fenitrothion	--	Immunochromatographic strip consists of NaYF_4_: Yb, Er-2, 4-D-IgG-fenitrothion-IgG	Portable sensors are prepared with unquestionable specificity	--	5	--	--	[175]
	Chlorpyrifos	Balloonflower angelica	NaYF_4_: Yb, Tm-DA	Dopamine quinone quench FL of UCNPs through PEI and chlorpyrifos prevent oxidation of DA which recover UCNPs FL	1.0–10^3^	0.38	95.4–120.0	5.3–8.5	[72]
	Diazinon	Apples	NaGdF_4_: Yb, Tm-Cu^2+^/AChE	Based on an (AChE) modulated fluorescence “off−on−off” strategy	0.1–50	0.05 ng mL^−1^	93.2–102.1	5.7–8.3	[179]
	Diazinon	Tea, apples	NaYF_4_: Yb, Er/Graphene Oxide	π-π interaction between UCNPs and GO	0.05–500	0.023	86.06–104.92 86.03–95.87	3.43–4.85	[180]
Neonicotinoid insecticides	Imidacloprid	Water, Chinese cabbage, honey	NaYF_4_: Yb, Er/AuNPs	Dual signal of immunofluorescence improves the sensitivity and selectivity of imidacloprid.	1.39–335.81	0.79	78.1–97.9	3.4–11.2	[181]
	Acetamiprid	Paddy water, pears	NaYF_4_: Yb, Er/cDNA-MNPs/aptamer	They label base complementary DNA, amine-functionalized UCNPs combine with negatively-charged DNA through electrostatic interaction	0.89–114.18	650	78.2–103.5	2.6–10.9	[182]
		Apples, strawberry	NaYF_4_: Yb, Er@molecularly imprinted polymer (MIP)	Quenching UCNPs emission peak at 542nm and specific recognition of MIP	20–800	8.3	89.6–97.9	1.6–2.9	[183]
Pyrethroids pesticides	Deltamethrin	Grape, cabbage	NaYF_4_: Yb, Er@MNPs@MIPs	Difunctional materials ensure a high degree selectivity of deltamethrin and separation	10^3^ –10^6^	0.749	95.6–02% 91.8–05%	2.97–4.07 2.42–5.20	[184]
	Fenpropathrin, Cypermethrin, fenvalerate	Cucumber, cabbage, apples and pears	NaYF_4_@NaYF_4_: Yb, Er@NaYF_4_/aminoated polystyrene magnetic microspheres-antigen	Devices for detecting multiple targets and miniaturized readout devices	--	0.01 0.015 0.011	83.4–97.8	--	[185]
Carbamate pesticide	Carbaryl	Tea	NaErF_4_: Tm^3+^@NaYF_4_/polydopamine embedded sodium alginate hydrogel	UCNPS immobilized-sodium alginate hydrogel system realizes the true sense of field detectable	0.5–200	0.5	90.51–105.33	2.28–4.46	[186]
Benz- fungicide imidazole	CBZ	Apples, cucumber, matcha powder	NaGdF_4_: Yb, Er/MnO_2_	The aptamer can self-assemble on the MnO_2_ nanosheet surface to quenching UCNPs FL	0.1–5000	0.05	93.84–96.62 90.14–93.96 93.80–109.4	2.02–4.39 2.90–4.30 1.87–3.51	[187]
Rhodamine B		Opaque fishes	NaYF_4_: Yb, Er	Opaque fishes not absorb RB and it will be quenching UCNPs FL through FRET	--	--	--	--	[188]
Norfloxacin		Milk	NaYF_4_: Yb, Er	Comparing analysis results of NOR strips, QD FICS and UCNPs FICS and LOD of UCNPs is lowest.	--	0.5	--	--	[189]
	Kanamycin	Milk, tap water	NaGd/YF_4_: Yb, Er- aptamer/BHQ_3_-cDNA	Kanamycin disrupts the FRET between BHQ_3_ and UCNPs by pairing with aptamer	24.2–2.42 × 10^4^	9.15	87–109.6 95.6–108.8	--	[190]
	Gallic acid	Green tea, orange juice	NaErF_4_: Tm@SiO_2_@ZIF-8/TMB	The emission spectrum of NaErF_4_: Tm@SiO_2_@ZIF-8 has overlap with the absorption spectra of oxTMB	0–5103.6	59.542	98.4–105	0.6–2.3	[136]
Metal ion	Cu^2+^	--	NaYF_4_@NaYF_4_: Er, Yb@NaYF_4_/rhodamine B hydrazide (RBH)	The distance between Er^3+^and RHB can enhance FL of UCNPs to improve signal sensitivity	--	--	---	-	[191]
	Pb^2+^	Tea	NaYF_4_: Gd, Yb, Ho/MNPs-AuNPs	Addition base complementary recognition to electrostatic interaction to construct FRET sensing platform	2.05–114.8	0.4674	101.6–107.0	0.8–2.1	[57]
	Hg^2+^	Green tea	NaYF_4_: Yb, Er@NaYF_4_/AuNPs- cysteine	Fluorescence turns off-on-off to detect Hg^2+^	0.48–480	1.08	93–102	1.57–2.06	[192]
		Ribbon fish	T-NaYF_4_: Yb, Tm	UCNP-T-Hg^2+^-T-UCNP reticular architecture on the surface of the electrode can accumulation of UCNP	2.01 × 10^−3^–0.201	8.04 × 10^−5^	--	--	[193]
IAcid ion	F	Milk	NaYF_4_: Yb, Er, Tm/curcumin	The specific recognition of curcumin with fluoride ions caused the shift in the UCNPs characteristic peak	475–3.8 × 10^3^ 95–2.18 × 10^4^	475	79.58–134.02%	0.94–22.11	[194]
	HSO_3_^−^	Sugar	NaYF_4_: Yb, Er@NaYF_4_/cyanine dye	NOBF_4_ functionalized UCNPs to electrostatic adsorption with cyanine dyes	81–9720	5.67	99.9–103.8	--	[195]

Neonicotinoid insecticides have persistence and high solubility, and they can affect the nervous system. Imidacloprid is a type of neonicotinoid insecticide that attracts numerous researchers to analyze its environmental residual. Si et al. [144] set a homogeneous immunoassay, and the analysis signal is derived from IFE between UCNPs and AuNPs. UCNPs are coupled to the antibody against imidacloprid, and AuNPs are used to label the antigen of imidacloprid. The competitive immunoreaction occurred between imidacloprid and antigen-AuNPs binding to antibody-UCNPs. If this method can be applied to polystyrene micro-well plates, the corresponding microplate readers should be developed to enable high-throughput screening. Sun et al. [145] successfully detected acetamiprid in rice, apple, pear, wheat, and cucumber using an acetamiprid aptamer-modified magnetic nanoparticle UCNPs fluorescent probe, and the detection limit is 0.65 μg L^−1^. Yu et al. [146] studied that because antibodies are susceptible to interference by organic solvents and other components in food, and the chemical stability of molecular imprinting is well known. They studied UCNPs@SiO2-MIP to detect acetamiprid in apples and strawberries, and the LOD is 8.3 ng mL^−1^.

In this review, the application of UCNPs in the detection of organic reagents in food in recent years is introduced in chronological order. Hu et al. [188] prepared polyethylene glycol hybrid ligand NaLuF_4_: Yb^3+^ and Er^3+^, and determined Rhodenine B and sodium fluorescein content in living opaque fish according to the position and intensity of its emission peak and luminescence resonant energy transfer between the fluorescent materials. Rong et al. [196] prepared a sensing platform consists of PEI-NaYF_4_: Yb, Er and GSH coupled with RBD to detect acrylamide. This study’s application can make food safety analysis faster and simpler. Wang et al. [197] determined the quality of red wine by analyzing the tannic acid content in red wine. They designed three different conjugated structures UCNPs: UCNPs@GDN, UCNPs@SO_3_H, UCNPs@PO(OH)_2_ were used to verify the efficiency of electron transfer between the material and tannic acid, and the results after transfer, respectively, through single, pairwise mixing and three mixed experiments to judge tannic acid. The content of tannic acid was also determined. In addition, the array calculation method successfully identified a variety of red wines. Hu et al. [189] made an immune chromatography test paper tag (antibody colloidal gold-quantum dots-UCNPs) for the visual judgment of milk, with different concentrations of norfloxacin, in the analysis of the sensitivity of a variety of fluorescent materials added, which can also compare quantum dots and the conversion of functional strength. The experiments show that quantum dots are the most sensitive of norfloxacin, while the UCNPs provide the most accurate analysis results in the milk. In order to detect heterocyclic aromatic amine carcinogens in fried meat products, Huang et al. [198] used NaYF_4_: Yb, Er grafted with anti-2-amino-3-methylimidazo [4, 5-f] quinoline (IQ) antibodies as fluorescent sensor through immunofluorescence sensing to detect IQ, and the LOD was as low as 7 ng L^−1^.

Currently, UCNPs are being widely used to detect active components such as enzymes in organisms, which is a mighty popular application. However, it is difficult to detect the enzyme in food because detection becomes difficult with changes in the environment. Detection becomes difficult. del Barrio et al. [199] successfully detected glucose oxidase in fruit juice using near-infrared-excited Tm^3+^ and Yb^3+^ to prepare fluorohemiflurate and pyrene fluorescein-conjugated graphene oxide mixed and coated on glass casting. This material has upconversion properties which should be more widely used in many more studies.

Inorganic ion residues in food can lead to reduced immunity and imbalance in trace element levels, etc. Qualitative and quantitative analysis of metal ions and inorganic salts in food is also particularly important. Zhang et al. [191] planed a FRET system including NaYF_4_@NaYF_4_: Er^3+^/Yb^3+^@NaYF_4_, which exhibit characteristic green emissions UCNPs and rhodamine B hydrazide (RBH), which is nonfluorescent. Adding Cu^2+^ to this system, a luminescence decrease in the nanoparticles occurs due to the energy transfer from UCNPs to RBH. Moreover, the structure design is in favor of improving sensitivity for Cu^2+^ detection and enhancing UCL detection signal. Chen et al. [57] used aptamer and FRET immunological recognition between NaYF_4_: Gd, Yb, Ho, and magnetic Fe_3_O_4_-modified (MNPs) GNPs, which generates a specific response to Pb^2+^ for dual recognition. It is sensitive to obtaining lead ions’ content in tea, and the detection limit is as low as 5.7 nmol L^−1^.

Fluoride ion is helpful for dental care and treatment of osteoporosis, while intaking excessively has negative active impacts on human health. Liu et al. [194] utilized IFE between amino-modified Yb^3+^, Er^3+^, Tm^3+^, co-doped NaYF_4_, and curcumin-F^−^ complex. The addition of F^−^ could cause a bathochromic shift in the maximum UV absorption peak of curcumin occurs and leads to the IFE-quenched fluorescence of UCNPs the large absorption bathochromic shift. Additionally, this results in a color change, which can be observed as well. This sensor has a fabulous response to F^−^ and has been successfully applied to milk products. It has an outstanding application prospect.

### 5.2. Analysis of Medical Sample

Synthetic drug molecules have played a decisive role in the history of human health. However, the residues of drugs in treating diseases and the number of residues should be of immediate concern. The application of the UCNPs in drug analysis has also been part of the research. Of course, if this fluorescence sensing platform can be combined with the real-time monitoring of people’s lives during the administration of drugs, it will be a future direction for the development of medicine and fluorescence analysis.

**Figure 7:**
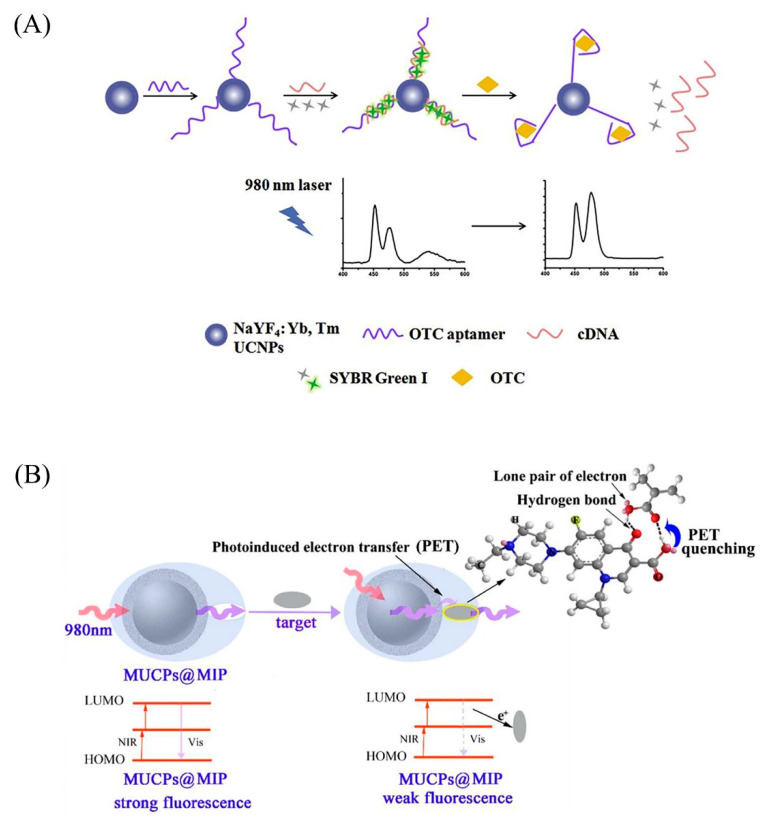
(**A**) Schematic illustration of the sensitive aptasensor for oxytetracycline based on upconversion luminescence resonance energy transfer. (**B**) Schematic diagram of quinolones fluorescence quenching the UCNPs mechanism. Reprinted with permission from [60,65]. Copyright 2015 and 2018, Elsevier.

The fluorescence of lanthanum (III) complexes produced by LRET from core–shell UCNPs is sensitive to drug molecules. Zhang et al. [65] imported SYBR Green-I as an energy donor to establish an LRET system with UCNPs to detect oxytetracycline. On the one hand, SYBR Green-I shows very week fluorescence unbinding with dsDNA. On the other hand, oxytetracycline stops combining with dsDNA and SYBR Green-I, then recovering UCNPs luminescence. This sensor is susceptible to LOD as low as 0.054 ng mL^−1^ (Figure 7A).

UCNPs are used to combine with precious metals such as gold and silver nanoparticles using Surface-enhanced Raman scattering (SERS) technology. At the same time, the SERS enhancement degree has extreme dependence on the excitation wavelength, and long-time exposure to the object under test under the irradiation of ultraviolet, visible laser power loss will have a certain degree. The near-infrared excitation of UCNPs will help it. Ma et al. [200] made NaYF_4_: Yb, Er@SiO_2_@Ag, which significantly improved the Raman enhancement ability of silver nanoparticles. Additionally, it was used to detect methylamphetamine successfully. It provides a new method for drug analysis.

In order to use quick and convenient fluorescence spectroscopy to analyze drugs, molecularly imprinted polymers (MIPs) are always used in combination with up-conversion nanoparticles with low background fluorescence due to their excellent selectivity. Tang et al. [60] synthesized layer-by-layer compounds of MIP and Fe_3_O_4_ nanoparticles on NaYF_4_: Yb^3+^, Er^3+^particles (MUCPs@MIP). They tested multiple drugs by using different quinolones as templates. Moreover, core–shell MIPs are designed to overcome some drawbacks such as the nonuniform distribution of binding sites, uncompleted removal of template or leakage, slow mass transfer, irregular morphology, etc. They are also designed to enhance recognition efficiency (Figure 7B).

Immunofluorescence analysis is also a commonly used analytical method, Zhang et al. [201] designed a lateral flow immunochromatographic and UCNPs to detect triamcinolone acetonide (TCA) cosmetics. Immunochromatography combined with fluorescence spectrum essentially solves the problems of narrow linear range, low sensitivity, and rough quantitative results. Additionally, the LOD for TCA in a cosmetic sample is 20 μg kg^−1^.

Drug anti-counterfeiting is an innovation in the application of UCNPs in medical science; a smart phone identification technology based on an upconversion fluorescent 3D quick response code for tracking and anti-counterfeiting of a drug was developed by You et al. [73]. They were resolving some drawbacks in existing anti-counterfeit technology such as high cost, complex fabrication process, and sophisticated operation as well. Multilayer inkjet printing and segmentation will also increase the amount of information stored per unit area. QR contains drug information also. Therefore, an upconversion fluorescent-based 3D QR code is promising as a powerful tool for drug anti-counterfeiting.

## 6. Conclusions and Perspectives

Basing on the special fluorescent properties of the UCNPs, the progress of research on one synthesis and analysis application of the UCNPs was reported in this review. The applications of UCNPs probes for analytical detection in environmental science, bioscience, food science, and medical science were summarized.

The application of the UCNPs in the field of analysis had attracted the attention of more and more researchers. Additionally, future research will focus on how to better utilize the excellent properties of the UCNPs in anticipation of solving the applications that have emerged, such as its weak detection signal, weak specificity, low detection range, and it not being widely used for real-time detection problems:

I. Different testing environments set different conditions for researchers, affecting the performance of UCNPs applications. So, the improvement in the luminescence efficiency of UCNPs according to the different analytical environments is a question worthy of deep pondering.

II. Designing practical UCNPs analysis platforms for different target analytes remains one of the challenges of applied research: (i) Depending on the type of substance to be measured, such as inorganic ions, small organic molecules, biological macromolecules, etc., the design of the UCNPs analysis platform with high matching to the properties of the substance to be measured to improve the selectivity is a challenge to be overcome in future integrated substance analysis. (ii) The study of easy functionalization of the UCNPs has already been applied in the field of analysis. In the following research to take more targeted functionalization of the UCNPs. According to the nature of the substances to be measured, the scope of application of the UCNPs in analytical chemistry becomes broader. Additionally, it will become another hot issue in the field of the UCNPs analysis in the future.

III. From the applicated materials, test methods, analysis conditions, test environment, and other aspects of the study on the impact of various factors on the test results, dedication to improving the efficiency of the analysis is also the future of the analysis process, thus requiring researchers to explore the direction of their efforts.

Based on the many desirable properties of the UCNPs mentioned in the paper and the wide range of applications of the UCNPs in the analytical field, they will essentially solve most of the challenging problems faced in today’s applications as the research progresses.

## Data Availability

Not applicable.
